# Supplementary material for: Prediction of antiviral drugs against African swine fever viruses based on protein–protein interaction analysis
Source: PeerJ. 2020 Apr 1;8:e8855. doi: 10.7717/peerj.8855 (PMC7127483; doi:10.7717/peerj.8855)

Supplementary Figures

Figure S1. Contribution energy for each residue in the template (A) and modelled complex between Geldanamycin and swine HSP90AB1 (B). MM: contains van der Waals, electrostatic interactions, and net non-bonded potential energy between the protein and inhibitor; Polar: polar solvation energy; Apolar: non-polar solvation energy; Total: total energy.

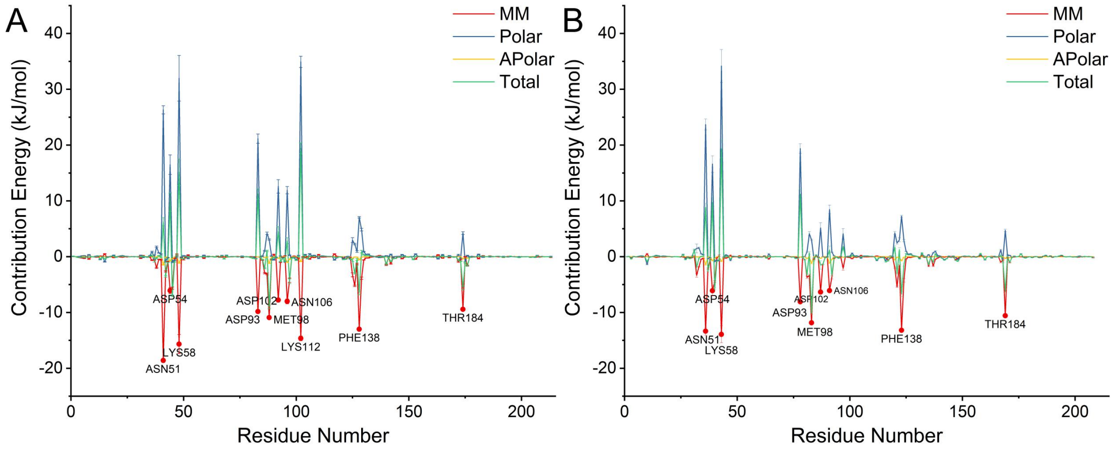

Supplement: Supplemental Information 1 — MM: contains Van der Waals, electrostatic interactions, and net non-bonded potential energy between the protein and inhibitor; Polar: polar solvation energy; Apolar: non-polar solvation energy; Total: total energy. [file peerj-08-8855-s001.pdf]
