# Supplementary material for: Prediction of antiviral drugs against African swine fever viruses based on protein–protein interaction analysis
Source: PeerJ. 2020 Apr 1;8:e8855. doi: 10.7717/peerj.8855 (PMC7127483; doi:10.7717/peerj.8855)

Figure S2. Binding energy of receptor-ligand complex for the template (A) and modelled complex between Geldanamycin and swine HSP90AB1 (B) during MD simulations (10 ns). ΔE_{vdw} refer to van der Waal energy, ΔE_{elec} refer to electrostatic energy, ΔE_{mm} refer to total potential energy between the protein and ligand ($\Delta E_{mm} = \Delta E_{vdw} + \Delta E_{elec}$), ΔE_{pol} refer to polar solvation energy, ΔE_{apol} refer to non-polar solvation energy and $\Delta E_{binding}$ refer to the total binding energy ($\Delta E_{binding} = \Delta E_{mm} + \Delta E_{pol} + \Delta E_{apol}$).

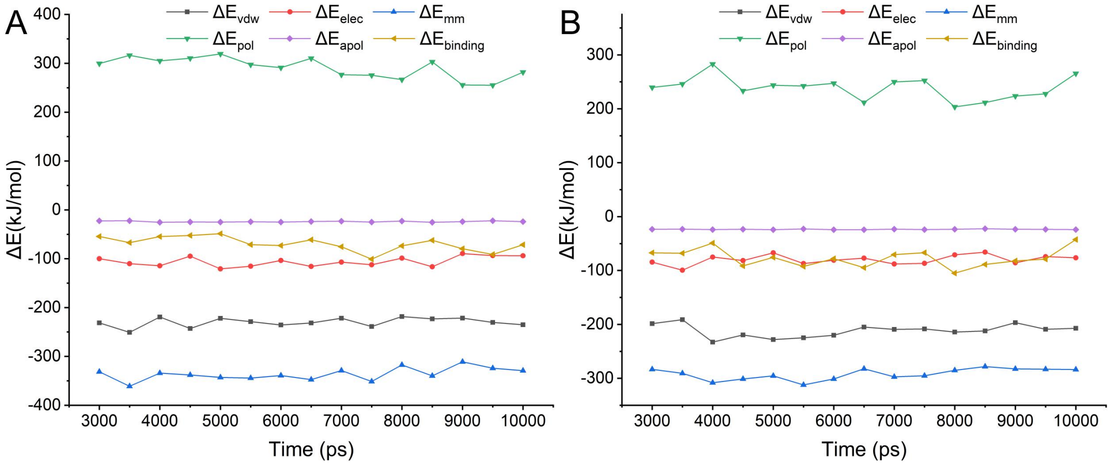

Supplement: Supplemental Information 2 — ΔEvdw refer to Van der Waal energy, ΔEelec refer to electrostatic energy, ΔEmm refer to total potential energy between the protein and ligand (ΔEmm = ΔEvdw + ΔEelec), ΔEpol refer to polar solvation energy, ΔEapol refer to non-polar solvation energy and ΔEbinding refer to the total binding energy (ΔEbinding = ΔEmm + ΔEpol + ΔEapol). [file peerj-08-8855-s002.pdf]
