# Supplementary material for: Prediction of antiviral drugs against African swine fever viruses based on protein–protein interaction analysis
Source: PeerJ. 2020 Apr 1;8:e8855. doi: 10.7717/peerj.8855 (PMC7127483; doi:10.7717/peerj.8855)
Supplement: Supplemental Information 3 [file peerj-08-8855-s003.doc]

**Supplementary Tables**

**Table S1.The curated protein-protein interactions between ASFV and swine.**

| **Swine protein** | | **ASFV protein** | **Source** | **Pubmed** |
| --- | --- | --- | --- | --- |
| **STRING ID** | **Gene symbol** |
| ENSSSCP00000000906 | HSP90B1 | DPOL | Viruses.STRING |  |
| ENSSSCP00000001172 | HIST1H2AA/LOC100153329 | DPOL | Viruses.STRING |  |
| ENSSSCP00000001238 | HIST1H2AJ/LOC100154071 | DPOL | Viruses.STRING |  |
| ENSSSCP00000001491 | TNF | A238L | Viruses.STRING |  |
| ENSSSCP00000001854 | HSP90AB1 | DPOL | Viruses.STRING |  |
| ENSSSCP00000002743 | HSP90AA1 | DPOL | Viruses.STRING |  |
| ENSSSCP00000006841 | LOC100156073 | DPOL | Viruses.STRING |  |
| ENSSSCP00000007112 | HIST2H2AC/LOC106510170 | DPOL | Viruses.STRING |  |
| ENSSSCP00000007115 | LOC102159655 | DPOL | Viruses.STRING |  |
| ENSSSCP00000008491 | TRAP1 | DPOL | Viruses.STRING |  |
| ENSSSCP00000013805 | RELA | A238L | Viruses.STRING |  |
| ENSSSCP00000014532 | PIN1 | DPOL | Viruses.STRING |  |
| ENSSSCP00000019487 | LOC100157763 | DPOL | Viruses.STRING |  |
| ENSSSCP00000020093 | H2AFX/LOC100522201 | DPOL | Viruses.STRING |  |
| ENSSSCP00000021537 | PIN4 | DPOL | Viruses.STRING |  |
| ENSSSCP00000024912 | FBXO2 | pF1055L | Viruses.STRING |  |
| ENSSSCP00000024912 | FBXO2 | TDK | Viruses.STRING |  |
| ENSSSCP00000024912 | FBXO2 | TMK | Viruses.STRING |  |
| ENSSSCP00000024932 | DNAJA3 | pF1055L | Viruses.STRING |  |
| ENSSSCP00000024932 | DNAJA3 | TMK | Viruses.STRING |  |
| ENSSSCP00000024932 | DNAJA3 | TDK | Viruses.STRING |  |
| ENSSSCP00000030691 | SNAPIN | pF1055L | Viruses.STRING |  |
| ENSSSCP00000030691 | SNAPIN | TMK | Viruses.STRING |  |
| ENSSSCP00000030691 | SNAPIN | TDK | Viruses.STRING |  |
| ENSSSCP00000030691 | SNAPIN | TDK | Homology-method |  |
| ENSSSCP00000024912 | FBXO2 | TDK | Homology-method |  |
| ENSSSCP00000024932 | DNAJA3 | TDK | Homology-method |  |
| ENSSSCP00000008635 | IL1B2 | L83L | Literature | 29605728 |
| ENSSSCP00000029600 | NACA | j4R | Literature | 12208975 |
| ENSSSCP00000010570 | DYNLL1 | p54 | Literature | 20686048 |
| ENSSSCP00000009105 | PPP1CB | DP71L | Literature | 20702639 |
| ENSSSCP00000010492 | PPP1CC | DP71L | Literature | 20702639 |
| ENSSSCP00000013730 | PPP1CA | DP71L | Literature | 20702639 |
| ENSSSCP00000016724 | CASP3 | A224L | Literature | 12805900 |
| ENSSSCP00000009781 | PPP3CA | A238L | Literature | 11000210 |
| ENSSSCP00000017711 | DBNL | CD2v | Literature | 14718626 |
| ENSSSCP00000030229 | BAG6 | EP152R | Literature | 27497620 |
| ENSSSCP00000014315 | SGTA | EP152R | Literature | 27497620 |
| ENSSSCP00000025047 | XPO1 | p37 | Literature | 15331706 |
| ENSSSCP00000000817 | BID | A179L | Literature | 18329683 |
| ENSSSCP00000001645 | BAK1 | A179L | Literature | 18329683 |
| ENSSSCP00000003404 | BAX | A179L | Literature | 18329683 |
| ENSSSCP00000026450 | HNRNPK | p30 | Literature | 29978065 |
| - | RPL23A | g5Rp | Literature | 29021398 |
