# Supplementary material for: Prediction of antiviral drugs against African swine fever viruses based on protein–protein interaction analysis
Source: PeerJ. 2020 Apr 1;8:e8855. doi: 10.7717/peerj.8855 (PMC7127483; doi:10.7717/peerj.8855)
Supplement: Supplemental Information 4 [file peerj-08-8855-s004.doc]

Table S2.The enriched GO terms and KEGG pathways for ASFV-interacting swine proteins and ASFV infection-associated proteins.

| **Part I: Enriched GO terms in the domain of Cellular Component for ASFV-interacting swine proteins .** | | | | | | | |
| --- | --- | --- | --- | --- | --- | --- | --- |
| **GO term** | **Description** | **GeneRatio** | **BgRatio** | **pvalue** | **p.adjust** | **qvalue** | **Count** |
| GO:0004721 | phosphoprotein phosphatase activity | 4/19 | 41/5087 | 1.29E-05 | 0.001096821 | 0.000814975 | 4 |
| GO:0016791 | phosphatase activity | 4/19 | 73/5087 | 0.000128552 | 0.005463467 | 0.004059542 | 4 |
| GO:0051082 | unfolded protein binding | 3/19 | 35/5087 | 0.000268173 | 0.00642713 | 0.004775576 | 3 |
| GO:0042578 | phosphoric ester hydrolase activity | 4/19 | 91/5087 | 0.000302453 | 0.00642713 | 0.004775576 | 4 |
|  |  |  |  |  |  |  |  |
| **Part II: Enriched KEGG pathways for ASFV-interacting swine proteins.** | | | | | | | |
| **GO term** | **Description** | **GeneRatio** | **BgRatio** | **pvalue** | **p.adjust** | **qvalue** | **Count** |
| ssc04217 | Necroptosis | 13/30 | 147/7714 | 2.32E-15 | 3.20E-13 | 1.05E-13 | 13 |
| ssc05034 | Alcoholism | 10/30 | 147/7714 | 1.01E-10 | 6.95E-09 | 2.28E-09 | 10 |
| ssc05322 | Systemic lupus erythematosus | 8/30 | 96/7714 | 2.00E-09 | 9.21E-08 | 3.02E-08 | 8 |
| ssc04657 | IL-17 signaling pathway | 7/30 | 88/7714 | 3.25E-08 | 1.12E-06 | 3.68E-07 | 7 |
| ssc05168 | Herpes simplex infection | 8/30 | 172/7714 | 2.00E-07 | 5.52E-06 | 1.81E-06 | 8 |
| ssc05014 | Amyotrophic lateral sclerosis (ALS) | 5/30 | 48/7714 | 9.55E-07 | 2.17E-05 | 7.13E-06 | 5 |
| ssc05163 | Human cytomegalovirus infection | 8/30 | 215/7714 | 1.10E-06 | 2.17E-05 | 7.13E-06 | 8 |
| ssc05152 | Tuberculosis | 7/30 | 161/7714 | 2.06E-06 | 3.55E-05 | 1.16E-05 | 7 |
| ssc04215 | Apoptosis - multiple species | 4/30 | 32/7714 | 6.20E-06 | 9.50E-05 | 3.12E-05 | 4 |
| ssc05170 | Human immunodeficiency virus 1 infection | 7/30 | 201/7714 | 8.99E-06 | 0.000119051 | 3.90E-05 | 7 |
| ssc04210 | Apoptosis | 6/30 | 134/7714 | 1.04E-05 | 0.000119051 | 3.90E-05 | 6 |
| ssc05418 | Fluid shear stress and atherosclerosis | 6/30 | 134/7714 | 1.04E-05 | 0.000119051 | 3.90E-05 | 6 |
| ssc05160 | Hepatitis C | 6/30 | 146/7714 | 1.69E-05 | 0.000179693 | 5.89E-05 | 6 |
| ssc04621 | NOD-like receptor signaling pathway | 6/30 | 148/7714 | 1.83E-05 | 0.000180335 | 5.91E-05 | 6 |
| ssc04932 | Non-alcoholic fatty liver disease (NAFLD) | 6/30 | 150/7714 | 1.98E-05 | 0.000181701 | 5.96E-05 | 6 |
| ssc04141 | Protein processing in endoplasmic reticulum | 6/30 | 161/7714 | 2.95E-05 | 0.000254626 | 8.35E-05 | 6 |
| ssc04933 | AGE-RAGE signaling pathway in diabetic complications | 5/30 | 100/7714 | 3.65E-05 | 0.000296153 | 9.71E-05 | 5 |
| ssc04659 | Th17 cell differentiation | 5/30 | 104/7714 | 4.41E-05 | 0.00033796 | 0.000110849 | 5 |
| ssc05134 | Legionellosis | 4/30 | 53/7714 | 4.77E-05 | 0.00034635 | 0.000113601 | 4 |
| ssc05167 | Kaposi sarcoma-associated herpesvirus infection | 6/30 | 177/7714 | 5.04E-05 | 0.000347441 | 0.000113959 | 6 |
| ssc04931 | Insulin resistance | 5/30 | 109/7714 | 5.52E-05 | 0.000363046 | 0.000119077 | 5 |
| ssc05203 | Viral carcinogenesis | 6/30 | 183/7714 | 6.07E-05 | 0.000380678 | 0.00012486 | 6 |
| ssc05169 | Epstein-Barr virus infection | 6/30 | 193/7714 | 8.16E-05 | 0.000489836 | 0.000160663 | 6 |
| ssc04720 | Long-term potentiation | 4/30 | 64/7714 | 0.000100441 | 0.000554435 | 0.000181851 | 4 |
| ssc05031 | Amphetamine addiction | 4/30 | 64/7714 | 0.000100441 | 0.000554435 | 0.000181851 | 4 |
| ssc01524 | Platinum drug resistance | 4/30 | 69/7714 | 0.000134829 | 0.000715631 | 0.000234723 | 4 |
| ssc05133 | Pertussis | 4/30 | 73/7714 | 0.00016793 | 0.000858308 | 0.00028152 | 4 |
| ssc01523 | Antifolate resistance | 3/30 | 32/7714 | 0.000244008 | 0.001202613 | 0.00039445 | 3 |
| ssc04218 | Cellular senescence | 5/30 | 154/7714 | 0.000282887 | 0.001346151 | 0.000441529 | 5 |
| ssc05161 | Hepatitis B | 5/30 | 156/7714 | 0.000300359 | 0.00138165 | 0.000453173 | 5 |
| ssc05222 | Small cell lung cancer | 4/30 | 91/7714 | 0.000392798 | 0.0017323 | 0.000568184 | 4 |
| ssc05146 | Amoebiasis | 4/30 | 92/7714 | 0.000409546 | 0.0017323 | 0.000568184 | 4 |
| ssc05010 | Alzheimer disease | 5/30 | 168/7714 | 0.000423081 | 0.0017323 | 0.000568184 | 5 |
| ssc05215 | Prostate cancer | 4/30 | 93/7714 | 0.000426799 | 0.0017323 | 0.000568184 | 4 |
| ssc04750 | Inflammatory mediator regulation of TRP channels | 4/30 | 98/7714 | 0.000520955 | 0.002054049 | 0.000673716 | 4 |
| ssc04650 | Natural killer cell mediated cytotoxicity | 4/30 | 100/7714 | 0.000562466 | 0.002156118 | 0.000707194 | 4 |
| ssc04625 | C-type lectin receptor signaling pathway | 4/30 | 102/7714 | 0.000606283 | 0.002261272 | 0.000741683 | 4 |
| ssc04668 | TNF signaling pathway | 4/30 | 104/7714 | 0.000652479 | 0.00236953 | 0.000777191 | 4 |
| ssc05205 | Proteoglycans in cancer | 5/30 | 196/7714 | 0.000854759 | 0.003024532 | 0.000992028 | 5 |
| ssc04114 | Oocyte meiosis | 4/30 | 113/7714 | 0.000891742 | 0.003076508 | 0.001009076 | 4 |
| ssc04071 | Sphingolipid signaling pathway | 4/30 | 117/7714 | 0.001015748 | 0.003418859 | 0.001121365 | 4 |
| ssc04380 | Osteoclast differentiation | 4/30 | 124/7714 | 0.001261274 | 0.004144185 | 0.001359267 | 4 |
| ssc04728 | Dopaminergic synapse | 4/30 | 126/7714 | 0.001338448 | 0.004233019 | 0.001388404 | 4 |
| ssc05166 | Human T-cell leukemia virus 1 infection | 5/30 | 217/7714 | 0.001349658 | 0.004233019 | 0.001388404 | 5 |
| ssc04612 | Antigen processing and presentation | 3/30 | 59/7714 | 0.001490014 | 0.004569377 | 0.001498728 | 3 |
| ssc05321 | Inflammatory bowel disease (IBD) | 3/30 | 62/7714 | 0.001719877 | 0.00515963 | 0.001692327 | 3 |
| ssc04622 | RIG-I-like receptor signaling pathway | 3/30 | 63/7714 | 0.00180115 | 0.005288482 | 0.00173459 | 3 |
| ssc05140 | Leishmaniasis | 3/30 | 65/7714 | 0.001970826 | 0.005666125 | 0.001858454 | 3 |
| ssc04115 | p53 signaling pathway | 3/30 | 69/7714 | 0.002339384 | 0.006588471 | 0.002160978 | 3 |
| ssc04921 | Oxytocin signaling pathway | 4/30 | 148/7714 | 0.002418376 | 0.006674717 | 0.002189266 | 4 |
| ssc05212 | Pancreatic cancer | 3/30 | 72/7714 | 0.002642102 | 0.007149216 | 0.002344899 | 3 |
| ssc05164 | Influenza A | 4/30 | 153/7714 | 0.002728801 | 0.007241817 | 0.002375272 | 4 |
| ssc05220 | Chronic myeloid leukemia | 3/30 | 74/7714 | 0.002856775 | 0.007438395 | 0.002439748 | 3 |
| ssc04022 | cGMP-PKG signaling pathway | 4/30 | 165/7714 | 0.003583842 | 0.009158709 | 0.003004001 | 4 |
|  |  |  |  |  |  |  |  |
| **Part III: Enriched GO terms in the domain of Biological Process for ASFV infection-associated proteins.** | | | | | | | |
| **GO term** | **Description** | **GeneRatio** | **BgRatio** | **pvalue** | **p.adjust** | **qvalue** | **Count** |
| GO:0006915 | apoptotic process | 158/995 | 394/4719 | 1.79E-19 | 5.62E-16 | 4.57E-16 | 158 |
| GO:0012501 | programmed cell death | 160/995 | 401/4719 | 1.84E-19 | 5.62E-16 | 4.57E-16 | 160 |
| GO:0008219 | cell death | 164/995 | 423/4719 | 1.82E-18 | 3.71E-15 | 3.02E-15 | 164 |
| GO:0010557 | positive regulation of macromolecule biosynthetic process | 140/995 | 345/4719 | 8.63E-18 | 1.32E-14 | 1.08E-14 | 140 |
| GO:0042981 | regulation of apoptotic process | 133/995 | 325/4719 | 2.80E-17 | 3.43E-14 | 2.79E-14 | 133 |
| GO:0043067 | regulation of programmed cell death | 134/995 | 329/4719 | 3.38E-17 | 3.44E-14 | 2.80E-14 | 134 |
| GO:0010941 | regulation of cell death | 138/995 | 350/4719 | 2.87E-16 | 2.51E-13 | 2.05E-13 | 138 |
| GO:0006468 | protein phosphorylation | 150/995 | 396/4719 | 8.03E-16 | 6.14E-13 | 5.00E-13 | 150 |
| GO:0045935 | positive regulation of nucleobase-containing compound metabolic process | 143/995 | 373/4719 | 1.31E-15 | 8.88E-13 | 7.23E-13 | 143 |
| GO:0016310 | phosphorylation | 168/995 | 470/4719 | 6.57E-15 | 4.02E-12 | 3.27E-12 | 168 |
| GO:0007049 | cell cycle | 128/995 | 327/4719 | 7.92E-15 | 4.41E-12 | 3.59E-12 | 128 |
| GO:0010628 | positive regulation of gene expression | 138/995 | 363/4719 | 1.00E-14 | 5.10E-12 | 4.15E-12 | 138 |
| GO:0009891 | positive regulation of biosynthetic process | 144/995 | 386/4719 | 1.50E-14 | 7.07E-12 | 5.75E-12 | 144 |
| GO:0000278 | mitotic cell cycle | 77/995 | 162/4719 | 2.01E-14 | 8.80E-12 | 7.17E-12 | 77 |
| GO:0051247 | positive regulation of protein metabolic process | 137/995 | 364/4719 | 3.24E-14 | 1.32E-11 | 1.08E-11 | 137 |
| GO:0031328 | positive regulation of cellular biosynthetic process | 141/995 | 379/4719 | 3.85E-14 | 1.47E-11 | 1.20E-11 | 141 |
| GO:0032270 | positive regulation of cellular protein metabolic process | 126/995 | 331/4719 | 1.55E-13 | 5.57E-11 | 4.54E-11 | 126 |
| GO:0022402 | cell cycle process | 96/995 | 230/4719 | 2.83E-13 | 9.62E-11 | 7.83E-11 | 96 |
| GO:0042127 | regulation of cell proliferation | 135/995 | 366/4719 | 3.18E-13 | 1.02E-10 | 8.33E-11 | 135 |
| GO:0051254 | positive regulation of RNA metabolic process | 116/995 | 302/4719 | 7.95E-13 | 2.43E-10 | 1.98E-10 | 116 |
| GO:0045893 | positive regulation of transcription, DNA-templated | 111/995 | 287/4719 | 1.55E-12 | 4.13E-10 | 3.36E-10 | 111 |
| GO:1902680 | positive regulation of RNA biosynthetic process | 111/995 | 287/4719 | 1.55E-12 | 4.13E-10 | 3.36E-10 | 111 |
| GO:1903508 | positive regulation of nucleic acid-templated transcription | 111/995 | 287/4719 | 1.55E-12 | 4.13E-10 | 3.36E-10 | 111 |
| GO:0071310 | cellular response to organic substance | 161/995 | 471/4719 | 2.26E-12 | 5.76E-10 | 4.69E-10 | 161 |
| GO:0065009 | regulation of molecular function | 159/995 | 467/4719 | 4.63E-12 | 1.13E-09 | 9.22E-10 | 159 |
| GO:0006366 | transcription from RNA polymerase II promoter | 141/995 | 400/4719 | 5.06E-12 | 1.19E-09 | 9.70E-10 | 141 |
| GO:0044093 | positive regulation of molecular function | 109/995 | 285/4719 | 5.86E-12 | 1.33E-09 | 1.08E-09 | 109 |
| GO:0033554 | cellular response to stress | 135/995 | 381/4719 | 1.01E-11 | 2.21E-09 | 1.80E-09 | 135 |
| GO:0051276 | chromosome organization | 85/995 | 207/4719 | 2.08E-11 | 4.39E-09 | 3.57E-09 | 85 |
| GO:1903047 | mitotic cell cycle process | 61/995 | 131/4719 | 3.51E-11 | 7.16E-09 | 5.83E-09 | 61 |
| GO:0008283 | cell proliferation | 147/995 | 432/4719 | 3.67E-11 | 7.24E-09 | 5.90E-09 | 147 |
| GO:0006457 | protein folding | 37/995 | 64/4719 | 1.19E-10 | 2.28E-08 | 1.85E-08 | 37 |
| GO:0051726 | regulation of cell cycle | 92/995 | 238/4719 | 1.57E-10 | 2.91E-08 | 2.37E-08 | 92 |
| GO:0051054 | positive regulation of DNA metabolic process | 31/995 | 49/4719 | 1.61E-10 | 2.91E-08 | 2.37E-08 | 31 |
| GO:0050790 | regulation of catalytic activity | 124/995 | 354/4719 | 1.92E-10 | 3.35E-08 | 2.73E-08 | 124 |
| GO:0051052 | regulation of DNA metabolic process | 41/995 | 76/4719 | 2.27E-10 | 3.85E-08 | 3.14E-08 | 41 |
| GO:0006357 | regulation of transcription from RNA polymerase II promoter | 129/995 | 375/4719 | 3.14E-10 | 5.19E-08 | 4.22E-08 | 129 |
| GO:0006954 | inflammatory response | 70/995 | 169/4719 | 8.68E-10 | 1.40E-07 | 1.14E-07 | 70 |
| GO:0033993 | response to lipid | 62/995 | 143/4719 | 9.38E-10 | 1.47E-07 | 1.20E-07 | 62 |
| GO:0010647 | positive regulation of cell communication | 113/995 | 321/4719 | 9.71E-10 | 1.49E-07 | 1.21E-07 | 113 |
| GO:0048584 | positive regulation of response to stimulus | 142/995 | 432/4719 | 1.35E-09 | 2.01E-07 | 1.64E-07 | 142 |
| GO:0023056 | positive regulation of signaling | 113/995 | 323/4719 | 1.48E-09 | 2.16E-07 | 1.76E-07 | 113 |
| GO:0009967 | positive regulation of signal transduction | 106/995 | 300/4719 | 2.69E-09 | 3.82E-07 | 3.11E-07 | 106 |
| GO:0045944 | positive regulation of transcription from RNA polymerase II promoter | 82/995 | 214/4719 | 2.75E-09 | 3.82E-07 | 3.11E-07 | 82 |
| GO:0043085 | positive regulation of catalytic activity | 83/995 | 220/4719 | 5.19E-09 | 7.07E-07 | 5.75E-07 | 83 |
| GO:0051130 | positive regulation of cellular component organization | 84/995 | 224/4719 | 5.84E-09 | 7.77E-07 | 6.33E-07 | 84 |
| GO:0051240 | positive regulation of multicellular organismal process | 117/995 | 345/4719 | 6.17E-09 | 8.04E-07 | 6.54E-07 | 117 |
| GO:0051301 | cell division | 43/995 | 90/4719 | 1.14E-08 | 1.46E-06 | 1.19E-06 | 43 |
| GO:0031399 | regulation of protein modification process | 125/995 | 380/4719 | 1.45E-08 | 1.82E-06 | 1.48E-06 | 125 |
| GO:0007017 | microtubule-based process | 57/995 | 135/4719 | 1.48E-08 | 1.82E-06 | 1.48E-06 | 57 |
| GO:0023014 | signal transduction by protein phosphorylation | 70/995 | 181/4719 | 2.73E-08 | 3.28E-06 | 2.67E-06 | 70 |
| GO:0008284 | positive regulation of cell proliferation | 74/995 | 196/4719 | 3.57E-08 | 4.20E-06 | 3.42E-06 | 74 |
| GO:0010952 | positive regulation of peptidase activity | 24/995 | 39/4719 | 4.37E-08 | 5.05E-06 | 4.11E-06 | 24 |
| GO:0007167 | enzyme linked receptor protein signaling pathway | 78/995 | 212/4719 | 5.55E-08 | 6.29E-06 | 5.12E-06 | 78 |
| GO:0010950 | positive regulation of endopeptidase activity | 23/995 | 37/4719 | 6.42E-08 | 7.14E-06 | 5.82E-06 | 23 |
| GO:0071495 | cellular response to endogenous stimulus | 89/995 | 253/4719 | 7.17E-08 | 7.83E-06 | 6.37E-06 | 89 |
| GO:0009719 | response to endogenous stimulus | 97/995 | 283/4719 | 7.76E-08 | 8.34E-06 | 6.79E-06 | 97 |
| GO:0000165 | MAPK cascade | 67/995 | 175/4719 | 8.71E-08 | 9.19E-06 | 7.48E-06 | 67 |
| GO:0042325 | regulation of phosphorylation | 109/995 | 330/4719 | 1.07E-07 | 1.10E-05 | 8.99E-06 | 109 |
| GO:0010862 | positive regulation of pathway-restricted SMAD protein phosphorylation | 15/995 | 19/4719 | 1.08E-07 | 1.10E-05 | 8.99E-06 | 15 |
| GO:0007346 | regulation of mitotic cell cycle | 48/995 | 112/4719 | 1.21E-07 | 1.20E-05 | 9.77E-06 | 48 |
| GO:0071396 | cellular response to lipid | 43/995 | 96/4719 | 1.22E-07 | 1.20E-05 | 9.77E-06 | 43 |
| GO:0031401 | positive regulation of protein modification process | 88/995 | 252/4719 | 1.25E-07 | 1.22E-05 | 9.92E-06 | 88 |
| GO:0006259 | DNA metabolic process | 77/995 | 214/4719 | 2.00E-07 | 1.91E-05 | 1.56E-05 | 77 |
| GO:0000226 | microtubule cytoskeleton organization | 43/995 | 98/4719 | 2.50E-07 | 2.35E-05 | 1.91E-05 | 43 |
| GO:0050793 | regulation of developmental process | 144/995 | 473/4719 | 2.78E-07 | 2.52E-05 | 2.05E-05 | 144 |
| GO:0060389 | pathway-restricted SMAD protein phosphorylation | 16/995 | 22/4719 | 2.80E-07 | 2.52E-05 | 2.05E-05 | 16 |
| GO:0060393 | regulation of pathway-restricted SMAD protein phosphorylation | 16/995 | 22/4719 | 2.80E-07 | 2.52E-05 | 2.05E-05 | 16 |
| GO:0002224 | toll-like receptor signaling pathway | 21/995 | 34/4719 | 2.86E-07 | 2.53E-05 | 2.06E-05 | 21 |
| GO:0051091 | positive regulation of DNA binding transcription factor activity | 31/995 | 62/4719 | 3.65E-07 | 3.19E-05 | 2.60E-05 | 31 |
| GO:0051094 | positive regulation of developmental process | 91/995 | 269/4719 | 4.00E-07 | 3.45E-05 | 2.80E-05 | 91 |
| GO:0046777 | protein autophosphorylation | 26/995 | 48/4719 | 4.31E-07 | 3.59E-05 | 2.93E-05 | 26 |
| GO:0048545 | response to steroid hormone | 26/995 | 48/4719 | 4.31E-07 | 3.59E-05 | 2.93E-05 | 26 |
| GO:0038083 | peptidyl-tyrosine autophosphorylation | 12/995 | 14/4719 | 4.35E-07 | 3.59E-05 | 2.93E-05 | 12 |
| GO:0043065 | positive regulation of apoptotic process | 47/995 | 113/4719 | 4.81E-07 | 3.87E-05 | 3.15E-05 | 47 |
| GO:0043068 | positive regulation of programmed cell death | 47/995 | 113/4719 | 4.81E-07 | 3.87E-05 | 3.15E-05 | 47 |
| GO:0051090 | regulation of DNA binding transcription factor activity | 38/995 | 84/4719 | 4.87E-07 | 3.87E-05 | 3.15E-05 | 38 |
| GO:0001932 | regulation of protein phosphorylation | 100/995 | 305/4719 | 5.65E-07 | 4.44E-05 | 3.61E-05 | 100 |
| GO:0071383 | cellular response to steroid hormone stimulus | 21/995 | 35/4719 | 5.74E-07 | 4.44E-05 | 3.62E-05 | 21 |
| GO:0050679 | positive regulation of epithelial cell proliferation | 25/995 | 46/4719 | 6.62E-07 | 5.07E-05 | 4.12E-05 | 25 |
| GO:0010942 | positive regulation of cell death | 48/995 | 118/4719 | 8.00E-07 | 6.02E-05 | 4.90E-05 | 48 |
| GO:0080134 | regulation of response to stress | 82/995 | 239/4719 | 8.06E-07 | 6.02E-05 | 4.90E-05 | 82 |
| GO:0071897 | DNA biosynthetic process | 17/995 | 26/4719 | 1.27E-06 | 9.34E-05 | 7.60E-05 | 17 |
| GO:0051338 | regulation of transferase activity | 68/995 | 191/4719 | 1.66E-06 | 0.000119592 | 9.74E-05 | 68 |
| GO:0042327 | positive regulation of phosphorylation | 74/995 | 213/4719 | 1.67E-06 | 0.000119592 | 9.74E-05 | 74 |
| GO:1902531 | regulation of intracellular signal transduction | 115/995 | 370/4719 | 1.68E-06 | 0.000119592 | 9.74E-05 | 115 |
| GO:0002237 | response to molecule of bacterial origin | 35/995 | 78/4719 | 1.75E-06 | 0.000123058 | 0.000100178 | 35 |
| GO:0030163 | protein catabolic process | 72/995 | 206/4719 | 1.80E-06 | 0.000123085 | 0.0001002 | 72 |
| GO:1902533 | positive regulation of intracellular signal transduction | 72/995 | 206/4719 | 1.80E-06 | 0.000123085 | 0.0001002 | 72 |
| GO:0043408 | regulation of MAPK cascade | 57/995 | 152/4719 | 1.81E-06 | 0.000123085 | 0.0001002 | 57 |
| GO:0097190 | apoptotic signaling pathway | 50/995 | 128/4719 | 1.99E-06 | 0.00013361 | 0.000108768 | 50 |
| GO:0006955 | immune response | 121/995 | 395/4719 | 2.02E-06 | 0.00013415 | 0.000109207 | 121 |
| GO:2000026 | regulation of multicellular organismal development | 109/995 | 348/4719 | 2.14E-06 | 0.000141103 | 0.000114868 | 109 |
| GO:0043401 | steroid hormone mediated signaling pathway | 15/995 | 22/4719 | 2.49E-06 | 0.000161894 | 0.000131793 | 15 |
| GO:2001056 | positive regulation of cysteine-type endopeptidase activity | 19/995 | 32/4719 | 2.51E-06 | 0.000161894 | 0.000131793 | 19 |
| GO:0001934 | positive regulation of protein phosphorylation | 72/995 | 208/4719 | 2.70E-06 | 0.000172107 | 0.000140107 | 72 |
| GO:0019220 | regulation of phosphate metabolic process | 118/995 | 386/4719 | 3.07E-06 | 0.000194003 | 0.000157932 | 118 |
| GO:0140014 | mitotic nuclear division | 25/995 | 49/4719 | 3.11E-06 | 0.00019447 | 0.000158312 | 25 |
| GO:0044770 | cell cycle phase transition | 36/995 | 83/4719 | 3.33E-06 | 0.000205915 | 0.000167629 | 36 |
| GO:0030518 | intracellular steroid hormone receptor signaling pathway | 14/995 | 20/4719 | 3.39E-06 | 0.000207376 | 0.000168818 | 14 |
| GO:0051174 | regulation of phosphorus metabolic process | 118/995 | 387/4719 | 3.55E-06 | 0.000214873 | 0.000174921 | 118 |
| GO:0002221 | pattern recognition receptor signaling pathway | 21/995 | 38/4719 | 3.69E-06 | 0.000219162 | 0.000178413 | 21 |
| GO:0002758 | innate immune response-activating signal transduction | 21/995 | 38/4719 | 3.69E-06 | 0.000219162 | 0.000178413 | 21 |
| GO:0090100 | positive regulation of transmembrane receptor protein serine/threonine kinase signaling pathway | 18/995 | 30/4719 | 3.77E-06 | 0.000221929 | 0.000180666 | 18 |
| GO:0010605 | negative regulation of macromolecule metabolic process | 129/995 | 432/4719 | 4.01E-06 | 0.000233479 | 0.000190068 | 129 |
| GO:0070201 | regulation of establishment of protein localization | 56/995 | 152/4719 | 4.24E-06 | 0.000244773 | 0.000199262 | 56 |
| GO:1904951 | positive regulation of establishment of protein localization | 37/995 | 87/4719 | 4.30E-06 | 0.000245697 | 0.000200015 | 37 |
| GO:0042542 | response to hydrogen peroxide | 13/995 | 18/4719 | 4.48E-06 | 0.000253678 | 0.000206511 | 13 |
| GO:0052547 | regulation of peptidase activity | 31/995 | 68/4719 | 4.55E-06 | 0.000255712 | 0.000208167 | 31 |
| GO:0048468 | cell development | 125/995 | 417/4719 | 4.65E-06 | 0.000258937 | 0.000210792 | 125 |
| GO:0071103 | DNA conformation change | 25/995 | 50/4719 | 5.00E-06 | 0.00027506 | 0.000223918 | 25 |
| GO:0033044 | regulation of chromosome organization | 28/995 | 59/4719 | 5.03E-06 | 0.00027506 | 0.000223918 | 28 |
| GO:0051347 | positive regulation of transferase activity | 46/995 | 118/4719 | 5.46E-06 | 0.00029396 | 0.000239304 | 46 |
| GO:0006508 | proteolysis | 98/995 | 311/4719 | 5.48E-06 | 0.00029396 | 0.000239304 | 98 |
| GO:2001252 | positive regulation of chromosome organization | 20/995 | 36/4719 | 5.66E-06 | 0.000301225 | 0.000245218 | 20 |
| GO:0000070 | mitotic sister chromatid segregation | 15/995 | 23/4719 | 5.77E-06 | 0.000301753 | 0.000245648 | 15 |
| GO:0000086 | G2/M transition of mitotic cell cycle | 15/995 | 23/4719 | 5.77E-06 | 0.000301753 | 0.000245648 | 15 |
| GO:0034504 | protein localization to nucleus | 37/995 | 88/4719 | 5.94E-06 | 0.000307999 | 0.000250733 | 37 |
| GO:0002684 | positive regulation of immune system process | 73/995 | 216/4719 | 6.22E-06 | 0.000319926 | 0.000260442 | 73 |
| GO:0010564 | regulation of cell cycle process | 51/995 | 136/4719 | 6.37E-06 | 0.000324968 | 0.000264546 | 51 |
| GO:0032880 | regulation of protein localization | 67/995 | 194/4719 | 6.63E-06 | 0.000335296 | 0.000272954 | 67 |
| GO:0000280 | nuclear division | 34/995 | 79/4719 | 7.67E-06 | 0.000381865 | 0.000310865 | 34 |
| GO:0044772 | mitotic cell cycle phase transition | 34/995 | 79/4719 | 7.67E-06 | 0.000381865 | 0.000310865 | 34 |
| GO:0065004 | protein-DNA complex assembly | 26/995 | 54/4719 | 7.87E-06 | 0.000388507 | 0.000316271 | 26 |
| GO:0051302 | regulation of cell division | 19/995 | 34/4719 | 8.68E-06 | 0.000425032 | 0.000346006 | 19 |
| GO:0071407 | cellular response to organic cyclic compound | 36/995 | 86/4719 | 8.95E-06 | 0.000434582 | 0.00035378 | 36 |
| GO:0052548 | regulation of endopeptidase activity | 29/995 | 64/4719 | 1.07E-05 | 0.000516254 | 0.000420266 | 29 |
| GO:1901701 | cellular response to oxygen-containing compound | 62/995 | 178/4719 | 1.08E-05 | 0.000518318 | 0.000421947 | 62 |
| GO:0006913 | nucleocytoplasmic transport | 37/995 | 90/4719 | 1.11E-05 | 0.000521628 | 0.000424642 | 37 |
| GO:0051169 | nuclear transport | 37/995 | 90/4719 | 1.11E-05 | 0.000521628 | 0.000424642 | 37 |
| GO:0046031 | ADP metabolic process | 13/995 | 19/4719 | 1.14E-05 | 0.000532911 | 0.000433827 | 13 |
| GO:0007059 | chromosome segregation | 27/995 | 58/4719 | 1.17E-05 | 0.000532911 | 0.000433827 | 27 |
| GO:0071824 | protein-DNA complex subunit organization | 27/995 | 58/4719 | 1.17E-05 | 0.000532911 | 0.000433827 | 27 |
| GO:0070848 | response to growth factor | 50/995 | 135/4719 | 1.18E-05 | 0.000532911 | 0.000433827 | 50 |
| GO:0071363 | cellular response to growth factor stimulus | 50/995 | 135/4719 | 1.18E-05 | 0.000532911 | 0.000433827 | 50 |
| GO:0032846 | positive regulation of homeostatic process | 23/995 | 46/4719 | 1.20E-05 | 0.000539185 | 0.000438934 | 23 |
| GO:0050673 | epithelial cell proliferation | 39/995 | 97/4719 | 1.21E-05 | 0.000540388 | 0.000439914 | 39 |
| GO:0044839 | cell cycle G2/M phase transition | 15/995 | 24/4719 | 1.24E-05 | 0.000549881 | 0.000447641 | 15 |
| GO:0000819 | sister chromatid segregation | 18/995 | 32/4719 | 1.33E-05 | 0.000585828 | 0.000476904 | 18 |
| GO:0045595 | regulation of cell differentiation | 99/995 | 321/4719 | 1.37E-05 | 0.000598705 | 0.000487388 | 99 |
| GO:0050678 | regulation of epithelial cell proliferation | 34/995 | 81/4719 | 1.48E-05 | 0.000641389 | 0.000522135 | 34 |
| GO:0043549 | regulation of kinase activity | 58/995 | 165/4719 | 1.53E-05 | 0.000659172 | 0.000536611 | 58 |
| GO:0006096 | glycolytic process | 12/995 | 17/4719 | 1.56E-05 | 0.000662325 | 0.000539179 | 12 |
| GO:0006757 | ATP generation from ADP | 12/995 | 17/4719 | 1.56E-05 | 0.000662325 | 0.000539179 | 12 |
| GO:0009628 | response to abiotic stimulus | 59/995 | 169/4719 | 1.64E-05 | 0.000692188 | 0.000563489 | 59 |
| GO:0007051 | spindle organization | 20/995 | 38/4719 | 1.68E-05 | 0.000704549 | 0.000573552 | 20 |
| GO:0090092 | regulation of transmembrane receptor protein serine/threonine kinase signaling pathway | 26/995 | 56/4719 | 1.79E-05 | 0.000747228 | 0.000608296 | 26 |
| GO:0006266 | DNA ligation | 7/995 | 7/4719 | 1.82E-05 | 0.00075344 | 0.000613353 | 7 |
| GO:0006325 | chromatin organization | 51/995 | 141/4719 | 2.06E-05 | 0.000842787 | 0.000686087 | 51 |
| GO:0032496 | response to lipopolysaccharide | 30/995 | 69/4719 | 2.07E-05 | 0.000842787 | 0.000686087 | 30 |
| GO:0048285 | organelle fission | 36/995 | 89/4719 | 2.23E-05 | 0.000904703 | 0.000736491 | 36 |
| GO:0045859 | regulation of protein kinase activity | 55/995 | 156/4719 | 2.31E-05 | 0.000928497 | 0.000755861 | 55 |
| GO:0007169 | transmembrane receptor protein tyrosine kinase signaling pathway | 48/995 | 131/4719 | 2.44E-05 | 0.000977176 | 0.000795489 | 48 |
| GO:0002755 | MyD88-dependent toll-like receptor signaling pathway | 10/995 | 13/4719 | 2.55E-05 | 0.00101278 | 0.000824473 | 10 |
| GO:0009135 | purine nucleoside diphosphate metabolic process | 13/995 | 20/4719 | 2.64E-05 | 0.001022804 | 0.000832633 | 13 |
| GO:0009179 | purine ribonucleoside diphosphate metabolic process | 13/995 | 20/4719 | 2.64E-05 | 0.001022804 | 0.000832633 | 13 |
| GO:0009185 | ribonucleoside diphosphate metabolic process | 13/995 | 20/4719 | 2.64E-05 | 0.001022804 | 0.000832633 | 13 |
| GO:0051781 | positive regulation of cell division | 13/995 | 20/4719 | 2.64E-05 | 0.001022804 | 0.000832633 | 13 |
| GO:0007178 | transmembrane receptor protein serine/threonine kinase signaling pathway | 34/995 | 83/4719 | 2.75E-05 | 0.001057538 | 0.00086091 | 34 |
| GO:0090130 | tissue migration | 24/995 | 51/4719 | 2.85E-05 | 0.001090852 | 0.000888029 | 24 |
| GO:0002218 | activation of innate immune response | 21/995 | 42/4719 | 2.88E-05 | 0.001093656 | 0.000890312 | 21 |
| GO:0071216 | cellular response to biotic stimulus | 23/995 | 48/4719 | 2.91E-05 | 0.001098063 | 0.000893899 | 23 |
| GO:0031347 | regulation of defense response | 48/995 | 132/4719 | 3.07E-05 | 0.001154261 | 0.000939649 | 48 |
| GO:0043280 | positive regulation of cysteine-type endopeptidase activity involved in apoptotic process | 16/995 | 28/4719 | 3.12E-05 | 0.00116334 | 0.00094704 | 16 |
| GO:0051172 | negative regulation of nitrogen compound metabolic process | 118/995 | 404/4719 | 3.37E-05 | 0.001250018 | 0.001017602 | 118 |
| GO:0045184 | establishment of protein localization | 110/995 | 372/4719 | 3.58E-05 | 0.001318569 | 0.001073407 | 110 |
| GO:0001816 | cytokine production | 59/995 | 173/4719 | 3.64E-05 | 0.001333578 | 0.001085625 | 59 |
| GO:0031023 | microtubule organizing center organization | 14/995 | 23/4719 | 3.75E-05 | 0.001344942 | 0.001094877 | 14 |
| GO:0060395 | SMAD protein signal transduction | 14/995 | 23/4719 | 3.75E-05 | 0.001344942 | 0.001094877 | 14 |
| GO:0006165 | nucleoside diphosphate phosphorylation | 12/995 | 18/4719 | 3.78E-05 | 0.001344942 | 0.001094877 | 12 |
| GO:0046939 | nucleotide phosphorylation | 12/995 | 18/4719 | 3.78E-05 | 0.001344942 | 0.001094877 | 12 |
| GO:2000278 | regulation of DNA biosynthetic process | 12/995 | 18/4719 | 3.78E-05 | 0.001344942 | 0.001094877 | 12 |
| GO:0043066 | negative regulation of apoptotic process | 61/995 | 181/4719 | 4.03E-05 | 0.001425379 | 0.001160358 | 61 |
| GO:0007159 | leukocyte cell-cell adhesion | 33/995 | 81/4719 | 4.11E-05 | 0.001445227 | 0.001176515 | 33 |
| GO:1901700 | response to oxygen-containing compound | 81/995 | 258/4719 | 4.33E-05 | 0.001512738 | 0.001231474 | 81 |
| GO:0050870 | positive regulation of T cell activation | 23/995 | 49/4719 | 4.40E-05 | 0.001521633 | 0.001238715 | 23 |
| GO:0098813 | nuclear chromosome segregation | 23/995 | 49/4719 | 4.40E-05 | 0.001521633 | 0.001238715 | 23 |
| GO:0071219 | cellular response to molecule of bacterial origin | 20/995 | 40/4719 | 4.46E-05 | 0.001534117 | 0.001248878 | 20 |
| GO:0009057 | macromolecule catabolic process | 86/995 | 278/4719 | 4.59E-05 | 0.001570704 | 0.001278662 | 86 |
| GO:0000003 | reproduction | 91/995 | 298/4719 | 4.76E-05 | 0.00160864 | 0.001309544 | 91 |
| GO:0022414 | reproductive process | 91/995 | 298/4719 | 4.76E-05 | 0.00160864 | 0.001309544 | 91 |
| GO:0090087 | regulation of peptide transport | 54/995 | 156/4719 | 4.90E-05 | 0.001649201 | 0.001342564 | 54 |
| GO:0043069 | negative regulation of programmed cell death | 62/995 | 186/4719 | 5.07E-05 | 0.001694778 | 0.001379667 | 62 |
| GO:0051128 | regulation of cellular component organization | 136/995 | 482/4719 | 5.31E-05 | 0.001764281 | 0.001436248 | 136 |
| GO:0045786 | negative regulation of cell cycle | 42/995 | 113/4719 | 5.33E-05 | 0.001764281 | 0.001436248 | 42 |
| GO:0010629 | negative regulation of gene expression | 89/995 | 291/4719 | 5.43E-05 | 0.001785305 | 0.001453362 | 89 |
| GO:0007098 | centrosome cycle | 13/995 | 21/4719 | 5.60E-05 | 0.001814798 | 0.001477371 | 13 |
| GO:0045454 | cell redox homeostasis | 13/995 | 21/4719 | 5.60E-05 | 0.001814798 | 0.001477371 | 13 |
| GO:0000302 | response to reactive oxygen species | 16/995 | 29/4719 | 5.60E-05 | 0.001814798 | 0.001477371 | 16 |
| GO:0009892 | negative regulation of metabolic process | 137/995 | 487/4719 | 5.82E-05 | 0.001873897 | 0.001525483 | 137 |
| GO:0045785 | positive regulation of cell adhesion | 32/995 | 79/4719 | 6.14E-05 | 0.001965905 | 0.001600384 | 32 |
| GO:0030162 | regulation of proteolysis | 46/995 | 128/4719 | 6.29E-05 | 0.00200415 | 0.001631517 | 46 |
| GO:0033365 | protein localization to organelle | 59/995 | 176/4719 | 6.41E-05 | 0.002033259 | 0.001655214 | 59 |
| GO:0010638 | positive regulation of organelle organization | 49/995 | 139/4719 | 6.45E-05 | 0.002035655 | 0.001657164 | 49 |
| GO:1903039 | positive regulation of leukocyte cell-cell adhesion | 23/995 | 50/4719 | 6.55E-05 | 0.002055079 | 0.001672977 | 23 |
| GO:0030522 | intracellular receptor signaling pathway | 18/995 | 35/4719 | 6.68E-05 | 0.002085255 | 0.001697542 | 18 |
| GO:0006323 | DNA packaging | 22/995 | 47/4719 | 6.79E-05 | 0.002109992 | 0.00171768 | 22 |
| GO:0033674 | positive regulation of kinase activity | 38/995 | 100/4719 | 6.91E-05 | 0.002136932 | 0.001739611 | 38 |
| GO:0050707 | regulation of cytokine secretion | 20/995 | 41/4719 | 7.01E-05 | 0.002154915 | 0.001754251 | 20 |
| GO:0045740 | positive regulation of DNA replication | 10/995 | 14/4719 | 7.23E-05 | 0.002213269 | 0.001801755 | 10 |
| GO:0045862 | positive regulation of proteolysis | 30/995 | 73/4719 | 7.54E-05 | 0.002296527 | 0.001869533 | 30 |
| GO:0006397 | mRNA processing | 35/995 | 90/4719 | 7.67E-05 | 0.00230364 | 0.001875323 | 35 |
| GO:0006974 | cellular response to DNA damage stimulus | 59/995 | 177/4719 | 7.70E-05 | 0.00230364 | 0.001875323 | 59 |
| GO:0010562 | positive regulation of phosphorus metabolic process | 79/995 | 254/4719 | 7.72E-05 | 0.00230364 | 0.001875323 | 79 |
| GO:0045937 | positive regulation of phosphate metabolic process | 79/995 | 254/4719 | 7.72E-05 | 0.00230364 | 0.001875323 | 79 |
| GO:0007249 | I-kappaB kinase/NF-kappaB signaling | 26/995 | 60/4719 | 7.84E-05 | 0.002328357 | 0.001895444 | 26 |
| GO:0002682 | regulation of immune system process | 85/995 | 278/4719 | 8.12E-05 | 0.002394803 | 0.001949536 | 85 |
| GO:0031349 | positive regulation of defense response | 32/995 | 80/4719 | 8.20E-05 | 0.002394803 | 0.001949536 | 32 |
| GO:0005977 | glycogen metabolic process | 12/995 | 19/4719 | 8.30E-05 | 0.002394803 | 0.001949536 | 12 |
| GO:0006073 | cellular glucan metabolic process | 12/995 | 19/4719 | 8.30E-05 | 0.002394803 | 0.001949536 | 12 |
| GO:0044042 | glucan metabolic process | 12/995 | 19/4719 | 8.30E-05 | 0.002394803 | 0.001949536 | 12 |
| GO:0018108 | peptidyl-tyrosine phosphorylation | 29/995 | 70/4719 | 8.33E-05 | 0.002394803 | 0.001949536 | 29 |
| GO:0045597 | positive regulation of cell differentiation | 61/995 | 185/4719 | 8.33E-05 | 0.002394803 | 0.001949536 | 61 |
| GO:0051223 | regulation of protein transport | 50/995 | 144/4719 | 8.51E-05 | 0.002432557 | 0.00198027 | 50 |
| GO:0006183 | GTP biosynthetic process | 6/995 | 6/4719 | 8.68E-05 | 0.002448793 | 0.001993488 | 6 |
| GO:0032392 | DNA geometric change | 6/995 | 6/4719 | 8.68E-05 | 0.002448793 | 0.001993488 | 6 |
| GO:1901070 | guanosine-containing compound biosynthetic process | 6/995 | 6/4719 | 8.68E-05 | 0.002448793 | 0.001993488 | 6 |
| GO:0050727 | regulation of inflammatory response | 31/995 | 77/4719 | 9.15E-05 | 0.002567608 | 0.002090212 | 31 |
| GO:0032206 | positive regulation of telomere maintenance | 9/995 | 12/4719 | 9.43E-05 | 0.002610783 | 0.002125359 | 9 |
| GO:0048146 | positive regulation of fibroblast proliferation | 9/995 | 12/4719 | 9.43E-05 | 0.002610783 | 0.002125359 | 9 |
| GO:2000573 | positive regulation of DNA biosynthetic process | 9/995 | 12/4719 | 9.43E-05 | 0.002610783 | 0.002125359 | 9 |
| GO:0043406 | positive regulation of MAP kinase activity | 23/995 | 51/4719 | 9.58E-05 | 0.002642121 | 0.00215087 | 23 |
| GO:0015833 | peptide transport | 108/995 | 372/4719 | 9.81E-05 | 0.002691776 | 0.002191293 | 108 |
| GO:0043410 | positive regulation of MAPK cascade | 39/995 | 105/4719 | 9.98E-05 | 0.002726249 | 0.002219356 | 39 |
| GO:0071900 | regulation of protein serine/threonine kinase activity | 37/995 | 98/4719 | 0.000100752 | 0.002740449 | 0.002230916 | 37 |
| GO:0051092 | positive regulation of NF-kappaB transcription factor activity | 18/995 | 36/4719 | 0.000107548 | 0.002880235 | 0.002344711 | 18 |
| GO:0006333 | chromatin assembly or disassembly | 20/995 | 42/4719 | 0.000107614 | 0.002880235 | 0.002344711 | 20 |
| GO:0045787 | positive regulation of cell cycle | 32/995 | 81/4719 | 0.000108734 | 0.002880235 | 0.002344711 | 32 |
| GO:0000723 | telomere maintenance | 13/995 | 22/4719 | 0.000110597 | 0.002880235 | 0.002344711 | 13 |
| GO:0005976 | polysaccharide metabolic process | 13/995 | 22/4719 | 0.000110597 | 0.002880235 | 0.002344711 | 13 |
| GO:0009132 | nucleoside diphosphate metabolic process | 13/995 | 22/4719 | 0.000110597 | 0.002880235 | 0.002344711 | 13 |
| GO:0032200 | telomere organization | 13/995 | 22/4719 | 0.000110597 | 0.002880235 | 0.002344711 | 13 |
| GO:0034614 | cellular response to reactive oxygen species | 13/995 | 22/4719 | 0.000110597 | 0.002880235 | 0.002344711 | 13 |
| GO:0060281 | regulation of oocyte development | 13/995 | 22/4719 | 0.000110597 | 0.002880235 | 0.002344711 | 13 |
| GO:1905879 | regulation of oogenesis | 13/995 | 22/4719 | 0.000110597 | 0.002880235 | 0.002344711 | 13 |
| GO:0060548 | negative regulation of cell death | 65/995 | 202/4719 | 0.000111925 | 0.002899702 | 0.002360559 | 65 |
| GO:0031324 | negative regulation of cellular metabolic process | 123/995 | 435/4719 | 0.000113875 | 0.002899702 | 0.002360559 | 123 |
| GO:0032212 | positive regulation of telomere maintenance via telomerase | 8/995 | 10/4719 | 0.000113918 | 0.002899702 | 0.002360559 | 8 |
| GO:0035767 | endothelial cell chemotaxis | 8/995 | 10/4719 | 0.000113918 | 0.002899702 | 0.002360559 | 8 |
| GO:1904358 | positive regulation of telomere maintenance via telomere lengthening | 8/995 | 10/4719 | 0.000113918 | 0.002899702 | 0.002360559 | 8 |
| GO:0002791 | regulation of peptide secretion | 36/995 | 95/4719 | 0.000114188 | 0.002899702 | 0.002360559 | 36 |
| GO:0015031 | protein transport | 104/995 | 357/4719 | 0.000114851 | 0.002904487 | 0.002364454 | 104 |
| GO:0051603 | proteolysis involved in cellular protein catabolic process | 56/995 | 168/4719 | 0.000116931 | 0.002932865 | 0.002387556 | 56 |
| GO:0060284 | regulation of cell development | 56/995 | 168/4719 | 0.000116931 | 0.002932865 | 0.002387556 | 56 |
| GO:0010833 | telomere maintenance via telomere lengthening | 11/995 | 17/4719 | 0.000121737 | 0.003031022 | 0.002467462 | 11 |
| GO:0044257 | cellular protein catabolic process | 57/995 | 172/4719 | 0.000121835 | 0.003031022 | 0.002467462 | 57 |
| GO:0042886 | amide transport | 108/995 | 374/4719 | 0.000124476 | 0.003084183 | 0.002510739 | 108 |
| GO:0010720 | positive regulation of cell development | 33/995 | 85/4719 | 0.000127075 | 0.003135888 | 0.002552831 | 33 |
| GO:0022409 | positive regulation of cell-cell adhesion | 24/995 | 55/4719 | 0.000128708 | 0.003156911 | 0.002569945 | 24 |
| GO:0051345 | positive regulation of hydrolase activity | 37/995 | 99/4719 | 0.000128959 | 0.003156911 | 0.002569945 | 37 |
| GO:0006275 | regulation of DNA replication | 14/995 | 25/4719 | 0.000133333 | 0.003238092 | 0.002636032 | 14 |
| GO:0051225 | spindle assembly | 14/995 | 25/4719 | 0.000133333 | 0.003238092 | 0.002636032 | 14 |
| GO:0034097 | response to cytokine | 58/995 | 177/4719 | 0.000150752 | 0.003646639 | 0.002968617 | 58 |
| GO:0050863 | regulation of T cell activation | 29/995 | 72/4719 | 0.000151805 | 0.003657676 | 0.002977602 | 29 |
| GO:0017038 | protein import | 31/995 | 79/4719 | 0.000160779 | 0.003856443 | 0.003139413 | 31 |
| GO:1900182 | positive regulation of protein localization to nucleus | 16/995 | 31/4719 | 0.000161315 | 0.003856443 | 0.003139413 | 16 |
| GO:0008104 | protein localization | 137/995 | 497/4719 | 0.000165403 | 0.003920466 | 0.003191532 | 137 |
| GO:0051321 | meiotic cell cycle | 25/995 | 59/4719 | 0.000166089 | 0.003920466 | 0.003191532 | 25 |
| GO:0034599 | cellular response to oxidative stress | 19/995 | 40/4719 | 0.00016663 | 0.003920466 | 0.003191532 | 19 |
| GO:0006334 | nucleosome assembly | 17/995 | 34/4719 | 0.000167192 | 0.003920466 | 0.003191532 | 17 |
| GO:0071347 | cellular response to interleukin-1 | 12/995 | 20/4719 | 0.000167837 | 0.003920466 | 0.003191532 | 12 |
| GO:1900193 | regulation of oocyte maturation | 12/995 | 20/4719 | 0.000167837 | 0.003920466 | 0.003191532 | 12 |
| GO:0031497 | chromatin assembly | 18/995 | 37/4719 | 0.000168674 | 0.003925028 | 0.003195245 | 18 |
| GO:0045934 | negative regulation of nucleobase-containing compound metabolic process | 73/995 | 236/4719 | 0.000175468 | 0.004067676 | 0.003311371 | 73 |
| GO:0072594 | establishment of protein localization to organelle | 46/995 | 133/4719 | 0.000180356 | 0.004164079 | 0.00338985 | 46 |
| GO:0043122 | regulation of I-kappaB kinase/NF-kappaB signaling | 24/995 | 56/4719 | 0.000180988 | 0.004164079 | 0.00338985 | 24 |
| GO:0050708 | regulation of protein secretion | 32/995 | 83/4719 | 0.000186738 | 0.004280289 | 0.003484453 | 32 |
| GO:0006606 | protein import into nucleus | 27/995 | 66/4719 | 0.000187479 | 0.004281234 | 0.003485222 | 27 |
| GO:0018212 | peptidyl-tyrosine modification | 29/995 | 73/4719 | 0.000202147 | 0.004582 | 0.003730067 | 29 |
| GO:1903037 | regulation of leukocyte cell-cell adhesion | 29/995 | 73/4719 | 0.000202147 | 0.004582 | 0.003730067 | 29 |
| GO:0010594 | regulation of endothelial cell migration | 13/995 | 23/4719 | 0.000205613 | 0.004643355 | 0.003780013 | 13 |
| GO:0051222 | positive regulation of protein transport | 31/995 | 80/4719 | 0.000210612 | 0.004733931 | 0.003853749 | 31 |
| GO:0070997 | neuron death | 22/995 | 50/4719 | 0.00021117 | 0.004733931 | 0.003853749 | 22 |
| GO:0019221 | cytokine-mediated signaling pathway | 38/995 | 105/4719 | 0.000228688 | 0.005107915 | 0.004158198 | 38 |
| GO:0051336 | regulation of hydrolase activity | 51/995 | 153/4719 | 0.000234318 | 0.005214647 | 0.004245085 | 51 |
| GO:0046039 | GTP metabolic process | 9/995 | 13/4719 | 0.000249063 | 0.005504522 | 0.004481064 | 9 |
| GO:0009994 | oocyte differentiation | 15/995 | 29/4719 | 0.00025113 | 0.005504522 | 0.004481064 | 15 |
| GO:0032147 | activation of protein kinase activity | 24/995 | 57/4719 | 0.00025123 | 0.005504522 | 0.004481064 | 24 |
| GO:0051170 | nuclear import | 27/995 | 67/4719 | 0.00025217 | 0.005504522 | 0.004481064 | 27 |
| GO:0071902 | positive regulation of protein serine/threonine kinase activity | 27/995 | 67/4719 | 0.00025217 | 0.005504522 | 0.004481064 | 27 |
| GO:0034502 | protein localization to chromosome | 11/995 | 18/4719 | 0.00025364 | 0.005504522 | 0.004481064 | 11 |
| GO:1903538 | regulation of meiotic cell cycle process involved in oocyte maturation | 11/995 | 18/4719 | 0.00025364 | 0.005504522 | 0.004481064 | 11 |
| GO:0034728 | nucleosome organization | 18/995 | 38/4719 | 0.000258159 | 0.005582796 | 0.004544784 | 18 |
| GO:0043542 | endothelial cell migration | 16/995 | 32/4719 | 0.000260196 | 0.005607039 | 0.00456452 | 16 |
| GO:0045860 | positive regulation of protein kinase activity | 35/995 | 95/4719 | 0.00026969 | 0.005791235 | 0.004714468 | 35 |
| GO:0034976 | response to endoplasmic reticulum stress | 23/995 | 54/4719 | 0.00027466 | 0.005856872 | 0.004767901 | 23 |
| GO:2000116 | regulation of cysteine-type endopeptidase activity | 23/995 | 54/4719 | 0.00027466 | 0.005856872 | 0.004767901 | 23 |
| GO:0014070 | response to organic cyclic compound | 47/995 | 139/4719 | 0.00028178 | 0.005987834 | 0.004874513 | 47 |
| GO:0006112 | energy reserve metabolic process | 12/995 | 21/4719 | 0.000316963 | 0.006666031 | 0.005426613 | 12 |
| GO:0044264 | cellular polysaccharide metabolic process | 12/995 | 21/4719 | 0.000316963 | 0.006666031 | 0.005426613 | 12 |
| GO:0070555 | response to interleukin-1 | 12/995 | 21/4719 | 0.000316963 | 0.006666031 | 0.005426613 | 12 |
| GO:0010631 | epithelial cell migration | 21/995 | 48/4719 | 0.000322698 | 0.006737507 | 0.005484799 | 21 |
| GO:0090132 | epithelium migration | 21/995 | 48/4719 | 0.000322698 | 0.006737507 | 0.005484799 | 21 |
| GO:0030155 | regulation of cell adhesion | 46/995 | 136/4719 | 0.000323665 | 0.006737507 | 0.005484799 | 46 |
| GO:0005978 | glycogen biosynthetic process | 8/995 | 11/4719 | 0.000340335 | 0.006989421 | 0.005689874 | 8 |
| GO:0009250 | glucan biosynthetic process | 8/995 | 11/4719 | 0.000340335 | 0.006989421 | 0.005689874 | 8 |
| GO:0051310 | metaphase plate congression | 8/995 | 11/4719 | 0.000340335 | 0.006989421 | 0.005689874 | 8 |
| GO:0070498 | interleukin-1-mediated signaling pathway | 8/995 | 11/4719 | 0.000340335 | 0.006989421 | 0.005689874 | 8 |
| GO:0097191 | extrinsic apoptotic signaling pathway | 24/995 | 58/4719 | 0.000344463 | 0.007050555 | 0.005739642 | 24 |
| GO:0009205 | purine ribonucleoside triphosphate metabolic process | 33/995 | 89/4719 | 0.00034971 | 0.007134077 | 0.005807635 | 33 |
| GO:0032103 | positive regulation of response to external stimulus | 31/995 | 82/4719 | 0.000353244 | 0.007182228 | 0.005846833 | 31 |
| GO:0051704 | multi-organism process | 133/995 | 488/4719 | 0.000357702 | 0.007248797 | 0.005901024 | 133 |
| GO:1902850 | microtubule cytoskeleton organization involved in mitosis | 13/995 | 24/4719 | 0.000362737 | 0.007326563 | 0.005964331 | 13 |
| GO:1901214 | regulation of neuron death | 19/995 | 42/4719 | 0.000367663 | 0.007401632 | 0.006025443 | 19 |
| GO:0043405 | regulation of MAP kinase activity | 26/995 | 65/4719 | 0.000375745 | 0.00752178 | 0.006123251 | 26 |
| GO:0048144 | fibroblast proliferation | 10/995 | 16/4719 | 0.000381005 | 0.00752178 | 0.006123251 | 10 |
| GO:0048145 | regulation of fibroblast proliferation | 10/995 | 16/4719 | 0.000381005 | 0.00752178 | 0.006123251 | 10 |
| GO:0050000 | chromosome localization | 10/995 | 16/4719 | 0.000381005 | 0.00752178 | 0.006123251 | 10 |
| GO:0051303 | establishment of chromosome localization | 10/995 | 16/4719 | 0.000381005 | 0.00752178 | 0.006123251 | 10 |
| GO:1902749 | regulation of cell cycle G2/M phase transition | 10/995 | 16/4719 | 0.000381005 | 0.00752178 | 0.006123251 | 10 |
| GO:0051249 | regulation of lymphocyte activation | 34/995 | 93/4719 | 0.000388392 | 0.007642946 | 0.00622189 | 34 |
| GO:0048599 | oocyte development | 14/995 | 27/4719 | 0.000391483 | 0.007667473 | 0.006241856 | 14 |
| GO:0010632 | regulation of epithelial cell migration | 17/995 | 36/4719 | 0.000400238 | 0.007667473 | 0.006241856 | 17 |
| GO:0071222 | cellular response to lipopolysaccharide | 17/995 | 36/4719 | 0.000400238 | 0.007667473 | 0.006241856 | 17 |
| GO:0050729 | positive regulation of inflammatory response | 16/995 | 33/4719 | 0.000407447 | 0.007667473 | 0.006241856 | 16 |
| GO:2000113 | negative regulation of cellular macromolecule biosynthetic process | 72/995 | 238/4719 | 0.000407948 | 0.007667473 | 0.006241856 | 72 |
| GO:0006228 | UTP biosynthetic process | 5/995 | 5/4719 | 0.000413442 | 0.007667473 | 0.006241856 | 5 |
| GO:0046051 | UTP metabolic process | 5/995 | 5/4719 | 0.000413442 | 0.007667473 | 0.006241856 | 5 |
| GO:0051103 | DNA ligation involved in DNA repair | 5/995 | 5/4719 | 0.000413442 | 0.007667473 | 0.006241856 | 5 |
| GO:0070200 | establishment of protein localization to telomere | 5/995 | 5/4719 | 0.000413442 | 0.007667473 | 0.006241856 | 5 |
| GO:0070202 | regulation of establishment of protein localization to chromosome | 5/995 | 5/4719 | 0.000413442 | 0.007667473 | 0.006241856 | 5 |
| GO:0070203 | regulation of establishment of protein localization to telomere | 5/995 | 5/4719 | 0.000413442 | 0.007667473 | 0.006241856 | 5 |
| GO:0070230 | positive regulation of lymphocyte apoptotic process | 5/995 | 5/4719 | 0.000413442 | 0.007667473 | 0.006241856 | 5 |
| GO:0070486 | leukocyte aggregation | 5/995 | 5/4719 | 0.000413442 | 0.007667473 | 0.006241856 | 5 |
| GO:1903405 | protein localization to nuclear body | 5/995 | 5/4719 | 0.000413442 | 0.007667473 | 0.006241856 | 5 |
| GO:1904851 | positive regulation of establishment of protein localization to telomere | 5/995 | 5/4719 | 0.000413442 | 0.007667473 | 0.006241856 | 5 |
| GO:1904867 | protein localization to Cajal body | 5/995 | 5/4719 | 0.000413442 | 0.007667473 | 0.006241856 | 5 |
| GO:1904869 | regulation of protein localization to Cajal body | 5/995 | 5/4719 | 0.000413442 | 0.007667473 | 0.006241856 | 5 |
| GO:1904871 | positive regulation of protein localization to Cajal body | 5/995 | 5/4719 | 0.000413442 | 0.007667473 | 0.006241856 | 5 |
| GO:1990173 | protein localization to nucleoplasm | 5/995 | 5/4719 | 0.000413442 | 0.007667473 | 0.006241856 | 5 |
| GO:0080135 | regulation of cellular response to stress | 35/995 | 97/4719 | 0.000427126 | 0.007897313 | 0.006428962 | 35 |
| GO:0032101 | regulation of response to external stimulus | 54/995 | 168/4719 | 0.000435759 | 0.008019429 | 0.006528373 | 54 |
| GO:0050806 | positive regulation of synaptic transmission | 7/995 | 9/4719 | 0.000437662 | 0.008019429 | 0.006528373 | 7 |
| GO:0051653 | spindle localization | 7/995 | 9/4719 | 0.000437662 | 0.008019429 | 0.006528373 | 7 |
| GO:0009199 | ribonucleoside triphosphate metabolic process | 33/995 | 90/4719 | 0.000443002 | 0.008072942 | 0.006571936 | 33 |
| GO:0002764 | immune response-regulating signaling pathway | 27/995 | 69/4719 | 0.00044322 | 0.008072942 | 0.006571936 | 27 |
| GO:2001233 | regulation of apoptotic signaling pathway | 31/995 | 83/4719 | 0.000452506 | 0.008217625 | 0.006689718 | 31 |
| GO:0044089 | positive regulation of cellular component biogenesis | 36/995 | 101/4719 | 0.000465633 | 0.008430996 | 0.006863416 | 36 |
| GO:0018193 | peptidyl-amino acid modification | 75/995 | 251/4719 | 0.000468348 | 0.008431322 | 0.006863682 | 75 |
| GO:0010558 | negative regulation of macromolecule biosynthetic process | 74/995 | 247/4719 | 0.000468407 | 0.008431322 | 0.006863682 | 74 |
| GO:1903827 | regulation of cellular protein localization | 39/995 | 112/4719 | 0.000471678 | 0.008437086 | 0.006868375 | 39 |
| GO:0001817 | regulation of cytokine production | 51/995 | 157/4719 | 0.000472863 | 0.008437086 | 0.006868375 | 51 |
| GO:0071345 | cellular response to cytokine stimulus | 51/995 | 157/4719 | 0.000472863 | 0.008437086 | 0.006868375 | 51 |
| GO:1903537 | meiotic cell cycle process involved in oocyte maturation | 11/995 | 19/4719 | 0.000488299 | 0.0086421 | 0.00703527 | 11 |
| GO:0043933 | macromolecular complex subunit organization | 112/995 | 403/4719 | 0.000491755 | 0.0086421 | 0.00703527 | 112 |
| GO:0050663 | cytokine secretion | 20/995 | 46/4719 | 0.000492858 | 0.0086421 | 0.00703527 | 20 |
| GO:0051251 | positive regulation of lymphocyte activation | 26/995 | 66/4719 | 0.000498117 | 0.0086421 | 0.00703527 | 26 |
| GO:1903829 | positive regulation of cellular protein localization | 26/995 | 66/4719 | 0.000498117 | 0.0086421 | 0.00703527 | 26 |
| GO:0034134 | toll-like receptor 2 signaling pathway | 6/995 | 7/4719 | 0.000498474 | 0.0086421 | 0.00703527 | 6 |
| GO:0042026 | protein refolding | 6/995 | 7/4719 | 0.000498474 | 0.0086421 | 0.00703527 | 6 |
| GO:0051568 | histone H3-K4 methylation | 6/995 | 7/4719 | 0.000498474 | 0.0086421 | 0.00703527 | 6 |
| GO:0060291 | long-term synaptic potentiation | 6/995 | 7/4719 | 0.000498474 | 0.0086421 | 0.00703527 | 6 |
| GO:2000108 | positive regulation of leukocyte apoptotic process | 6/995 | 7/4719 | 0.000498474 | 0.0086421 | 0.00703527 | 6 |
| GO:0006090 | pyruvate metabolic process | 12/995 | 22/4719 | 0.000564602 | 0.009510056 | 0.007741846 | 12 |
| GO:0006470 | protein dephosphorylation | 12/995 | 22/4719 | 0.000564602 | 0.009510056 | 0.007741846 | 12 |
| GO:1903429 | regulation of cell maturation | 12/995 | 22/4719 | 0.000564602 | 0.009510056 | 0.007741846 | 12 |
| GO:0006302 | double-strand break repair | 18/995 | 40/4719 | 0.000566008 | 0.009510056 | 0.007741846 | 18 |
| GO:0007018 | microtubule-based movement | 18/995 | 40/4719 | 0.000566008 | 0.009510056 | 0.007741846 | 18 |
| GO:0000271 | polysaccharide biosynthetic process | 9/995 | 14/4719 | 0.000567185 | 0.009510056 | 0.007741846 | 9 |
| GO:0006278 | RNA-dependent DNA biosynthetic process | 9/995 | 14/4719 | 0.000567185 | 0.009510056 | 0.007741846 | 9 |
| GO:0007004 | telomere maintenance via telomerase | 9/995 | 14/4719 | 0.000567185 | 0.009510056 | 0.007741846 | 9 |
| GO:0010389 | regulation of G2/M transition of mitotic cell cycle | 9/995 | 14/4719 | 0.000567185 | 0.009510056 | 0.007741846 | 9 |
| GO:0010595 | positive regulation of endothelial cell migration | 9/995 | 14/4719 | 0.000567185 | 0.009510056 | 0.007741846 | 9 |
| GO:0070301 | cellular response to hydrogen peroxide | 9/995 | 14/4719 | 0.000567185 | 0.009510056 | 0.007741846 | 9 |
| GO:1904356 | regulation of telomere maintenance via telomere lengthening | 9/995 | 14/4719 | 0.000567185 | 0.009510056 | 0.007741846 | 9 |
| GO:0031098 | stress-activated protein kinase signaling cascade | 22/995 | 53/4719 | 0.000572707 | 0.009576406 | 0.00779586 | 22 |
| GO:0050776 | regulation of immune response | 52/995 | 162/4719 | 0.00057449 | 0.009580048 | 0.007798825 | 52 |
| GO:0008285 | negative regulation of cell proliferation | 47/995 | 143/4719 | 0.000583645 | 0.009686901 | 0.007885811 | 47 |
| GO:0044703 | multi-organism reproductive process | 62/995 | 201/4719 | 0.000584063 | 0.009686901 | 0.007885811 | 62 |
| GO:0060341 | regulation of cellular localization | 53/995 | 166/4719 | 0.000588712 | 0.009737617 | 0.007927097 | 53 |
| GO:0007254 | JNK cascade | 17/995 | 37/4719 | 0.000597068 | 0.0098492 | 0.008017933 | 17 |
|  |  |  |  |  |  |  |  |
| **Part IV: Enriched GO terms in the domain of Cellular Component for the ASFV infection-associated proteins.** | | | | | | | |
| **GO term** | **Description** | **GeneRatio** | **BgRatio** | **pvalue** | **p.adjust** | **qvalue** | **Count** |
| GO:0005654 | nucleoplasm | 162/1030 | 496/5431 | 1.34E-14 | 9.86E-12 | 7.91E-12 | 162 |
| GO:0015630 | microtubule cytoskeleton | 80/1030 | 190/5431 | 5.79E-14 | 2.13E-11 | 1.71E-11 | 80 |
| GO:0005856 | cytoskeleton | 117/1030 | 356/5431 | 6.79E-11 | 1.67E-08 | 1.34E-08 | 117 |
| GO:0005694 | chromosome | 75/1030 | 197/5431 | 1.36E-10 | 2.50E-08 | 2.01E-08 | 75 |
| GO:1902494 | catalytic complex | 126/1030 | 399/5431 | 2.34E-10 | 3.44E-08 | 2.76E-08 | 126 |
| GO:0044427 | chromosomal part | 69/1030 | 182/5431 | 9.55E-10 | 1.09E-07 | 8.74E-08 | 69 |
| GO:0005815 | microtubule organizing center | 46/1030 | 102/5431 | 1.04E-09 | 1.09E-07 | 8.74E-08 | 46 |
| GO:0044430 | cytoskeletal part | 93/1030 | 275/5431 | 1.40E-09 | 1.28E-07 | 1.03E-07 | 93 |
| GO:0016604 | nuclear body | 49/1030 | 114/5431 | 2.22E-09 | 1.79E-07 | 1.44E-07 | 49 |
| GO:0071013 | catalytic step 2 spliceosome | 22/1030 | 33/5431 | 2.43E-09 | 1.79E-07 | 1.44E-07 | 22 |
| GO:0008287 | protein serine/threonine phosphatase complex | 14/1030 | 16/5431 | 5.92E-09 | 3.63E-07 | 2.91E-07 | 14 |
| GO:1903293 | phosphatase complex | 14/1030 | 16/5431 | 5.92E-09 | 3.63E-07 | 2.91E-07 | 14 |
| GO:0000775 | chromosome, centromeric region | 21/1030 | 32/5431 | 8.72E-09 | 4.94E-07 | 3.96E-07 | 21 |
| GO:0044451 | nucleoplasm part | 74/1030 | 216/5431 | 4.01E-08 | 2.11E-06 | 1.69E-06 | 74 |
| GO:0005681 | spliceosomal complex | 30/1030 | 62/5431 | 1.27E-07 | 6.21E-06 | 4.98E-06 | 30 |
| GO:0005813 | centrosome | 34/1030 | 75/5431 | 1.38E-07 | 6.37E-06 | 5.11E-06 | 34 |
| GO:0000793 | condensed chromosome | 17/1030 | 26/5431 | 2.60E-07 | 1.13E-05 | 9.03E-06 | 17 |
| GO:0005875 | microtubule associated complex | 18/1030 | 29/5431 | 3.64E-07 | 1.49E-05 | 1.19E-05 | 18 |
| GO:0098687 | chromosomal region | 29/1030 | 62/5431 | 5.12E-07 | 1.94E-05 | 1.55E-05 | 29 |
| GO:0022624 | proteasome accessory complex | 10/1030 | 11/5431 | 5.30E-07 | 1.94E-05 | 1.55E-05 | 10 |
| GO:0000502 | proteasome complex | 17/1030 | 27/5431 | 5.79E-07 | 1.94E-05 | 1.55E-05 | 17 |
| GO:1905369 | endopeptidase complex | 17/1030 | 27/5431 | 5.79E-07 | 1.94E-05 | 1.55E-05 | 17 |
| GO:0000776 | kinetochore | 15/1030 | 23/5431 | 1.43E-06 | 4.56E-05 | 3.66E-05 | 15 |
| GO:1905368 | peptidase complex | 17/1030 | 31/5431 | 8.40E-06 | 0.000257703 | 0.000206767 | 17 |
| GO:0005819 | spindle | 25/1030 | 56/5431 | 8.90E-06 | 0.000261935 | 0.000210162 | 25 |
| GO:0000228 | nuclear chromosome | 41/1030 | 118/5431 | 3.10E-05 | 0.000878879 | 0.000705165 | 41 |
| GO:0061695 | transferase complex, transferring phosphorus-containing groups | 30/1030 | 78/5431 | 4.17E-05 | 0.001137473 | 0.000912647 | 30 |
| GO:0016607 | nuclear speck | 24/1030 | 57/5431 | 4.41E-05 | 0.001158842 | 0.000929792 | 24 |
| GO:0005838 | proteasome regulatory particle | 7/1030 | 8/5431 | 5.80E-05 | 0.001471462 | 0.001180621 | 7 |
| GO:0044450 | microtubule organizing center part | 13/1030 | 23/5431 | 6.57E-05 | 0.001611293 | 0.001292814 | 13 |
| GO:0005635 | nuclear envelope | 30/1030 | 80/5431 | 7.28E-05 | 0.001727348 | 0.00138593 | 30 |
| GO:0044454 | nuclear chromosome part | 38/1030 | 111/5431 | 8.68E-05 | 0.001995299 | 0.001600919 | 38 |
| GO:0000922 | spindle pole | 13/1030 | 24/5431 | 0.000118581 | 0.002644719 | 0.002121978 | 13 |
| GO:0005643 | nuclear pore | 8/1030 | 11/5431 | 0.000156063 | 0.003378294 | 0.002710559 | 8 |
| GO:0005665 | DNA-directed RNA polymerase II, core complex | 7/1030 | 9/5431 | 0.000217976 | 0.004525915 | 0.003631348 | 7 |
| GO:0098552 | side of membrane | 32/1030 | 92/5431 | 0.000221376 | 0.004525915 | 0.003631348 | 32 |
| GO:0008540 | proteasome regulatory particle, base subcomplex | 5/1030 | 5/5431 | 0.000243425 | 0.00474604 | 0.003807964 | 5 |
| GO:0000794 | condensed nuclear chromosome | 9/1030 | 14/5431 | 0.00024504 | 0.00474604 | 0.003807964 | 9 |
| GO:0000159 | protein phosphatase type 2A complex | 6/1030 | 7/5431 | 0.000269811 | 0.004843429 | 0.003886104 | 6 |
| GO:0002199 | zona pellucida receptor complex | 6/1030 | 7/5431 | 0.000269811 | 0.004843429 | 0.003886104 | 6 |
| GO:0005686 | U2 snRNP | 6/1030 | 7/5431 | 0.000269811 | 0.004843429 | 0.003886104 | 6 |
| GO:0005814 | centriole | 11/1030 | 20/5431 | 0.000338102 | 0.005924831 | 0.004753762 | 11 |
| GO:0031234 | extrinsic component of cytoplasmic side of plasma membrane | 12/1030 | 23/5431 | 0.000346721 | 0.005934569 | 0.004761575 | 12 |
| GO:0000785 | chromatin | 38/1030 | 119/5431 | 0.000439156 | 0.007345885 | 0.005893938 | 38 |
| GO:0019897 | extrinsic component of plasma membrane | 14/1030 | 30/5431 | 0.00049597 | 0.008111872 | 0.006508525 | 14 |
|  |  |  |  |  |  |  |  |
| **Part V: Enriched GO terms in the domain of Molecular Function for the ASFV infection-associated proteins.** | | | | | | | |
| **GO term** | **Description** | **GeneRatio** | **BgRatio** | **pvalue** | **p.adjust** | **qvalue** | **Count** |
| GO:0005524 | ATP binding | 202/1090 | 419/5087 | 2.08E-37 | 1.74E-34 | 1.44E-34 | 202 |
| GO:0030554 | adenyl nucleotide binding | 204/1090 | 427/5087 | 4.64E-37 | 1.74E-34 | 1.44E-34 | 204 |
| GO:0032559 | adenyl ribonucleotide binding | 204/1090 | 427/5087 | 4.64E-37 | 1.74E-34 | 1.44E-34 | 204 |
| GO:0004672 | protein kinase activity | 114/1090 | 190/5087 | 5.42E-32 | 1.52E-29 | 1.26E-29 | 114 |
| GO:0016773 | phosphotransferase activity, alcohol group as acceptor | 119/1090 | 222/5087 | 4.51E-27 | 1.01E-24 | 8.41E-25 | 119 |
| GO:0016301 | kinase activity | 130/1090 | 256/5087 | 1.52E-26 | 2.85E-24 | 2.37E-24 | 130 |
| GO:0016772 | transferase activity, transferring phosphorus-containing groups | 140/1090 | 304/5087 | 3.59E-23 | 5.77E-21 | 4.79E-21 | 140 |
| GO:0004674 | protein serine/threonine kinase activity | 71/1090 | 120/5087 | 1.09E-19 | 1.52E-17 | 1.27E-17 | 71 |
| GO:0003700 | DNA binding transcription factor activity | 130/1090 | 334/5087 | 3.67E-14 | 4.59E-12 | 3.81E-12 | 130 |
| GO:0140110 | transcription regulator activity | 148/1090 | 402/5087 | 1.17E-13 | 1.32E-11 | 1.09E-11 | 148 |
| GO:0004713 | protein tyrosine kinase activity | 33/1090 | 46/5087 | 3.03E-13 | 3.10E-11 | 2.57E-11 | 33 |
| GO:0019199 | transmembrane receptor protein kinase activity | 25/1090 | 32/5087 | 1.05E-11 | 9.80E-10 | 8.13E-10 | 25 |
| GO:0003707 | steroid hormone receptor activity | 26/1090 | 35/5087 | 2.94E-11 | 2.54E-09 | 2.11E-09 | 26 |
| GO:0008083 | growth factor activity | 36/1090 | 62/5087 | 2.98E-10 | 2.40E-08 | 1.99E-08 | 36 |
| GO:0004714 | transmembrane receptor protein tyrosine kinase activity | 17/1090 | 22/5087 | 3.32E-08 | 2.48E-06 | 2.06E-06 | 17 |
| GO:0004715 | non-membrane spanning protein tyrosine kinase activity | 16/1090 | 20/5087 | 3.61E-08 | 2.53E-06 | 2.10E-06 | 16 |
| GO:0019903 | protein phosphatase binding | 20/1090 | 29/5087 | 4.83E-08 | 3.19E-06 | 2.65E-06 | 20 |
| GO:0019888 | protein phosphatase regulator activity | 19/1090 | 27/5087 | 6.36E-08 | 3.97E-06 | 3.30E-06 | 19 |
| GO:0005057 | signal transducer activity, downstream of receptor | 21/1090 | 32/5087 | 8.36E-08 | 4.95E-06 | 4.11E-06 | 21 |
| GO:0004702 | signal transducer, downstream of receptor, with serine/threonine kinase activity | 17/1090 | 23/5087 | 1.02E-07 | 5.45E-06 | 4.52E-06 | 17 |
| GO:0005160 | transforming growth factor beta receptor binding | 17/1090 | 23/5087 | 1.02E-07 | 5.45E-06 | 4.52E-06 | 17 |
| GO:0051082 | unfolded protein binding | 22/1090 | 35/5087 | 1.28E-07 | 6.56E-06 | 5.44E-06 | 22 |
| GO:0019208 | phosphatase regulator activity | 19/1090 | 28/5087 | 1.58E-07 | 7.74E-06 | 6.42E-06 | 19 |
| GO:0003755 | peptidyl-prolyl cis-trans isomerase activity | 14/1090 | 18/5087 | 5.10E-07 | 2.30E-05 | 1.90E-05 | 14 |
| GO:0016859 | cis-trans isomerase activity | 14/1090 | 18/5087 | 5.10E-07 | 2.30E-05 | 1.90E-05 | 14 |
| GO:0019902 | phosphatase binding | 21/1090 | 35/5087 | 7.69E-07 | 3.32E-05 | 2.76E-05 | 21 |
| GO:0005102 | receptor binding | 137/1090 | 454/5087 | 2.86E-06 | 0.000119038 | 9.88E-05 | 137 |
| GO:0004879 | nuclear receptor activity | 14/1090 | 20/5087 | 4.17E-06 | 0.000161562 | 0.000134055 | 14 |
| GO:0098531 | transcription factor activity, direct ligand regulated sequence-specific DNA binding | 14/1090 | 20/5087 | 4.17E-06 | 0.000161562 | 0.000134055 | 14 |
| GO:0005125 | cytokine activity | 51/1090 | 133/5087 | 5.03E-06 | 0.000188541 | 0.000156441 | 51 |
| GO:0017111 | nucleoside-triphosphatase activity | 79/1090 | 235/5087 | 6.45E-06 | 0.000233709 | 0.000193919 | 79 |
| GO:0005126 | cytokine receptor binding | 56/1090 | 156/5087 | 1.79E-05 | 0.000628427 | 0.000521433 | 56 |
| GO:0005003 | ephrin receptor activity | 7/1090 | 7/5087 | 2.04E-05 | 0.00069569 | 0.000577244 | 7 |
| GO:0070851 | growth factor receptor binding | 19/1090 | 36/5087 | 3.33E-05 | 0.001099888 | 0.000912625 | 19 |
| GO:0019901 | protein kinase binding | 42/1090 | 110/5087 | 3.86E-05 | 0.001240633 | 0.001029407 | 42 |
| GO:0000975 | regulatory region DNA binding | 66/1090 | 197/5087 | 4.26E-05 | 0.001294052 | 0.001073731 | 66 |
| GO:0001067 | regulatory region nucleic acid binding | 66/1090 | 197/5087 | 4.26E-05 | 0.001294052 | 0.001073731 | 66 |
| GO:0043565 | sequence-specific DNA binding | 86/1090 | 276/5087 | 6.63E-05 | 0.001930371 | 0.001601712 | 86 |
| GO:0016462 | pyrophosphatase activity | 79/1090 | 249/5087 | 6.70E-05 | 0.001930371 | 0.001601712 | 79 |
| GO:0003924 | GTPase activity | 41/1090 | 109/5087 | 7.12E-05 | 0.001999683 | 0.001659224 | 41 |
| GO:0016818 | hydrolase activity, acting on acid anhydrides, in phosphorus-containing anhydrides | 79/1090 | 251/5087 | 9.07E-05 | 0.002486926 | 0.002063511 | 79 |
| GO:0031072 | heat shock protein binding | 12/1090 | 19/5087 | 9.83E-05 | 0.002631352 | 0.002183347 | 12 |
| GO:0016817 | hydrolase activity, acting on acid anhydrides | 79/1090 | 252/5087 | 0.000105283 | 0.002752047 | 0.002283493 | 79 |
| GO:0004675 | transmembrane receptor protein serine/threonine kinase activity | 8/1090 | 10/5087 | 0.000128846 | 0.003291432 | 0.002731044 | 8 |
| GO:0019900 | kinase binding | 43/1090 | 119/5087 | 0.000140673 | 0.003513705 | 0.002915474 | 43 |
| GO:0000981 | RNA polymerase II transcription factor activity, sequence-specific DNA binding | 61/1090 | 187/5087 | 0.000194522 | 0.004753091 | 0.003943846 | 61 |
| GO:0019904 | protein domain specific binding | 40/1090 | 110/5087 | 0.000204129 | 0.004881713 | 0.004050569 | 40 |
| GO:0048018 | receptor ligand activity | 74/1090 | 239/5087 | 0.000267259 | 0.006258315 | 0.005192796 | 74 |
| GO:0044212 | transcription regulatory region DNA binding | 61/1090 | 190/5087 | 0.000317005 | 0.007271707 | 0.006033651 | 61 |
| GO:0003723 | RNA binding | 119/1090 | 423/5087 | 0.000395698 | 0.0088953 | 0.007380817 | 119 |
| GO:0035325 | Toll-like receptor binding | 5/1090 | 5/5087 | 0.000448423 | 0.009882893 | 0.008200265 | 5 |
|  |  |  |  |  |  |  |  |
| **Part VI: Enriched KEGG pathways for the ASFV infection-associated proteins.** | | | | | | | |
| **GO term** | **Description** | **GeneRatio** | **BgRatio** | **pvalue** | **p.adjust** | **qvalue** | **Count** |
| ssc04010 | MAPK signaling pathway | 140/1395 | 286/7674 | 8.36E-34 | 2.39E-31 | 1.03E-31 | 140 |
| ssc05161 | Hepatitis B | 85/1395 | 136/7674 | 7.91E-31 | 1.13E-28 | 4.87E-29 | 85 |
| ssc04210 | Apoptosis | 82/1395 | 134/7674 | 7.76E-29 | 7.40E-27 | 3.19E-27 | 82 |
| ssc04151 | PI3K-Akt signaling pathway | 143/1395 | 335/7674 | 1.29E-26 | 8.10E-25 | 3.49E-25 | 143 |
| ssc04668 | TNF signaling pathway | 68/1395 | 104/7674 | 1.42E-26 | 8.10E-25 | 3.49E-25 | 68 |
| ssc05166 | Human T-cell leukemia virus 1 infection | 107/1395 | 217/7674 | 2.81E-26 | 1.34E-24 | 5.76E-25 | 107 |
| ssc05212 | Pancreatic cancer | 54/1395 | 72/7674 | 6.02E-26 | 2.46E-24 | 1.06E-24 | 54 |
| ssc04068 | FoxO signaling pathway | 77/1395 | 130/7674 | 8.06E-26 | 2.88E-24 | 1.24E-24 | 77 |
| ssc04066 | HIF-1 signaling pathway | 61/1395 | 96/7674 | 5.69E-23 | 1.72E-21 | 7.41E-22 | 61 |
| ssc05160 | Hepatitis C | 79/1395 | 146/7674 | 6.02E-23 | 1.72E-21 | 7.41E-22 | 79 |
| ssc05164 | Influenza A | 81/1395 | 153/7674 | 1.12E-22 | 2.90E-21 | 1.25E-21 | 81 |
| ssc04931 | Insulin resistance | 64/1395 | 108/7674 | 1.16E-21 | 2.77E-20 | 1.19E-20 | 64 |
| ssc05142 | Chagas disease (American trypanosomiasis) | 62/1395 | 103/7674 | 1.59E-21 | 3.29E-20 | 1.42E-20 | 62 |
| ssc05418 | Fluid shear stress and atherosclerosis | 73/1395 | 134/7674 | 1.61E-21 | 3.29E-20 | 1.42E-20 | 73 |
| ssc05203 | Viral carcinogenesis | 89/1395 | 183/7674 | 1.77E-21 | 3.37E-20 | 1.45E-20 | 89 |
| ssc05167 | Kaposi sarcoma-associated herpesvirus infection | 87/1395 | 177/7674 | 2.06E-21 | 3.68E-20 | 1.58E-20 | 87 |
| ssc05215 | Prostate cancer | 58/1395 | 93/7674 | 2.64E-21 | 4.43E-20 | 1.91E-20 | 58 |
| ssc04114 | Oocyte meiosis | 64/1395 | 113/7674 | 3.31E-20 | 5.26E-19 | 2.26E-19 | 64 |
| ssc05169 | Epstein-Barr virus infection | 90/1395 | 193/7674 | 3.88E-20 | 5.84E-19 | 2.51E-19 | 90 |
| ssc05170 | Human immunodeficiency virus 1 infection | 92/1395 | 201/7674 | 7.17E-20 | 1.02E-18 | 4.41E-19 | 92 |
| ssc04910 | Insulin signaling pathway | 71/1395 | 135/7674 | 8.45E-20 | 1.15E-18 | 4.96E-19 | 71 |
| ssc05205 | Proteoglycans in cancer | 89/1395 | 196/7674 | 5.65E-19 | 7.34E-18 | 3.16E-18 | 89 |
| ssc05210 | Colorectal cancer | 53/1395 | 87/7674 | 6.58E-19 | 8.18E-18 | 3.52E-18 | 53 |
| ssc04625 | C-type lectin receptor signaling pathway | 58/1395 | 102/7674 | 1.54E-18 | 1.84E-17 | 7.91E-18 | 58 |
| ssc04933 | AGE-RAGE signaling pathway in diabetic complications | 56/1395 | 99/7674 | 8.48E-18 | 9.70E-17 | 4.18E-17 | 56 |
| ssc04064 | NF-kappa B signaling pathway | 53/1395 | 91/7674 | 1.11E-17 | 1.17E-16 | 5.03E-17 | 53 |
| ssc05162 | Measles | 65/1395 | 126/7674 | 1.17E-17 | 1.17E-16 | 5.03E-17 | 65 |
| ssc04722 | Neurotrophin signaling pathway | 62/1395 | 117/7674 | 1.18E-17 | 1.17E-16 | 5.03E-17 | 62 |
| ssc03040 | Spliceosome | 64/1395 | 123/7674 | 1.18E-17 | 1.17E-16 | 5.03E-17 | 64 |
| ssc04014 | Ras signaling pathway | 95/1395 | 226/7674 | 2.19E-17 | 2.08E-16 | 8.97E-17 | 95 |
| ssc04620 | Toll-like receptor signaling pathway | 54/1395 | 95/7674 | 2.46E-17 | 2.27E-16 | 9.76E-17 | 54 |
| ssc04914 | Progesterone-mediated oocyte maturation | 51/1395 | 88/7674 | 6.21E-17 | 5.43E-16 | 2.34E-16 | 51 |
| ssc05163 | Human cytomegalovirus infection | 91/1395 | 215/7674 | 6.27E-17 | 5.43E-16 | 2.34E-16 | 91 |
| ssc04660 | T cell receptor signaling pathway | 55/1395 | 100/7674 | 9.31E-17 | 7.83E-16 | 3.37E-16 | 55 |
| ssc01521 | EGFR tyrosine kinase inhibitor resistance | 47/1395 | 78/7674 | 1.22E-16 | 9.98E-16 | 4.30E-16 | 47 |
| ssc04218 | Cellular senescence | 72/1395 | 154/7674 | 2.08E-16 | 1.65E-15 | 7.11E-16 | 72 |
| ssc05218 | Melanoma | 43/1395 | 69/7674 | 4.24E-16 | 3.28E-15 | 1.41E-15 | 43 |
| ssc04110 | Cell cycle | 61/1395 | 121/7674 | 5.01E-16 | 3.77E-15 | 1.62E-15 | 61 |
| ssc04921 | Oxytocin signaling pathway | 69/1395 | 147/7674 | 6.92E-16 | 5.07E-15 | 2.18E-15 | 69 |
| ssc04659 | Th17 cell differentiation | 55/1395 | 104/7674 | 9.44E-16 | 6.75E-15 | 2.91E-15 | 55 |
| ssc04621 | NOD-like receptor signaling pathway | 68/1395 | 146/7674 | 1.85E-15 | 1.29E-14 | 5.56E-15 | 68 |
| ssc05214 | Glioma | 43/1395 | 71/7674 | 1.91E-15 | 1.30E-14 | 5.59E-15 | 43 |
| ssc04380 | Osteoclast differentiation | 61/1395 | 124/7674 | 2.21E-15 | 1.47E-14 | 6.33E-15 | 61 |
| ssc04350 | TGF-beta signaling pathway | 49/1395 | 88/7674 | 2.34E-15 | 1.49E-14 | 6.41E-15 | 49 |
| ssc04657 | IL-17 signaling pathway | 49/1395 | 88/7674 | 2.34E-15 | 1.49E-14 | 6.41E-15 | 49 |
| ssc05221 | Acute myeloid leukemia | 40/1395 | 64/7674 | 3.97E-15 | 2.47E-14 | 1.06E-14 | 40 |
| ssc04152 | AMPK signaling pathway | 59/1395 | 121/7674 | 1.07E-14 | 6.51E-14 | 2.80E-14 | 59 |
| ssc05145 | Toxoplasmosis | 53/1395 | 103/7674 | 1.43E-14 | 8.54E-14 | 3.68E-14 | 53 |
| ssc05152 | Tuberculosis | 71/1395 | 161/7674 | 1.47E-14 | 8.58E-14 | 3.70E-14 | 71 |
| ssc04015 | Rap1 signaling pathway | 83/1395 | 203/7674 | 1.69E-14 | 9.65E-14 | 4.16E-14 | 83 |
| ssc05168 | Herpes simplex infection | 74/1395 | 172/7674 | 1.98E-14 | 1.11E-13 | 4.78E-14 | 74 |
| ssc04211 | Longevity regulating pathway | 48/1395 | 89/7674 | 2.41E-14 | 1.32E-13 | 5.70E-14 | 48 |
| ssc04810 | Regulation of actin cytoskeleton | 83/1395 | 205/7674 | 3.27E-14 | 1.77E-13 | 7.60E-14 | 83 |
| ssc04012 | ErbB signaling pathway | 46/1395 | 84/7674 | 3.94E-14 | 2.09E-13 | 8.99E-14 | 46 |
| ssc05211 | Renal cell carcinoma | 40/1395 | 68/7674 | 7.38E-14 | 3.84E-13 | 1.65E-13 | 40 |
| ssc05220 | Chronic myeloid leukemia | 42/1395 | 74/7674 | 9.74E-14 | 4.97E-13 | 2.14E-13 | 42 |
| ssc03015 | mRNA surveillance pathway | 46/1395 | 86/7674 | 1.26E-13 | 6.34E-13 | 2.73E-13 | 46 |
| ssc05133 | Pertussis | 41/1395 | 73/7674 | 3.12E-13 | 1.54E-12 | 6.62E-13 | 41 |
| ssc05223 | Non-small cell lung cancer | 37/1395 | 64/7674 | 1.35E-12 | 6.56E-12 | 2.83E-12 | 37 |
| ssc04360 | Axon guidance | 73/1395 | 181/7674 | 1.52E-12 | 7.23E-12 | 3.11E-12 | 73 |
| ssc01522 | Endocrine resistance | 46/1395 | 91/7674 | 1.86E-12 | 8.71E-12 | 3.75E-12 | 46 |
| ssc05226 | Gastric cancer | 63/1395 | 147/7674 | 2.22E-12 | 1.02E-11 | 4.41E-12 | 63 |
| ssc04140 | Autophagy - animal | 57/1395 | 128/7674 | 3.75E-12 | 1.68E-11 | 7.24E-12 | 57 |
| ssc05224 | Breast cancer | 62/1395 | 145/7674 | 3.76E-12 | 1.68E-11 | 7.24E-12 | 62 |
| ssc04060 | Cytokine-cytokine receptor interaction | 93/1395 | 259/7674 | 4.19E-12 | 1.84E-11 | 7.93E-12 | 93 |
| ssc04261 | Adrenergic signaling in cardiomyocytes | 59/1395 | 136/7674 | 6.05E-12 | 2.62E-11 | 1.13E-11 | 59 |
| ssc04728 | Dopaminergic synapse | 56/1395 | 126/7674 | 6.41E-12 | 2.71E-11 | 1.17E-11 | 56 |
| ssc05213 | Endometrial cancer | 34/1395 | 58/7674 | 6.45E-12 | 2.71E-11 | 1.17E-11 | 34 |
| ssc04720 | Long-term potentiation | 36/1395 | 64/7674 | 8.33E-12 | 3.45E-11 | 1.49E-11 | 36 |
| ssc05230 | Central carbon metabolism in cancer | 34/1395 | 59/7674 | 1.26E-11 | 5.15E-11 | 2.22E-11 | 34 |
| ssc04071 | Sphingolipid signaling pathway | 52/1395 | 116/7674 | 2.47E-11 | 9.93E-11 | 4.28E-11 | 52 |
| ssc01524 | Platinum drug resistance | 37/1395 | 69/7674 | 2.92E-11 | 1.16E-10 | 5.00E-11 | 37 |
| ssc04530 | Tight junction | 66/1395 | 166/7674 | 3.89E-11 | 1.52E-10 | 6.56E-11 | 66 |
| ssc04926 | Relaxin signaling pathway | 55/1395 | 129/7674 | 7.23E-11 | 2.79E-10 | 1.20E-10 | 55 |
| ssc04217 | Necroptosis | 60/1395 | 147/7674 | 8.62E-11 | 3.29E-10 | 1.42E-10 | 60 |
| ssc03050 | Proteasome | 27/1395 | 44/7674 | 2.28E-10 | 8.47E-10 | 3.65E-10 | 27 |
| ssc04390 | Hippo signaling pathway | 60/1395 | 150/7674 | 2.29E-10 | 8.47E-10 | 3.65E-10 | 60 |
| ssc04922 | Glucagon signaling pathway | 44/1395 | 95/7674 | 2.31E-10 | 8.47E-10 | 3.65E-10 | 44 |
| ssc04213 | Longevity regulating pathway - multiple species | 33/1395 | 61/7674 | 2.54E-10 | 9.20E-10 | 3.96E-10 | 33 |
| ssc05031 | Amphetamine addiction | 34/1395 | 64/7674 | 2.61E-10 | 9.34E-10 | 4.02E-10 | 34 |
| ssc05165 | Human papillomavirus infection | 103/1395 | 318/7674 | 3.23E-10 | 1.14E-09 | 4.91E-10 | 103 |
| ssc04024 | cAMP signaling pathway | 72/1395 | 196/7674 | 3.71E-10 | 1.29E-09 | 5.57E-10 | 72 |
| ssc05134 | Legionellosis | 30/1395 | 53/7674 | 3.78E-10 | 1.30E-09 | 5.60E-10 | 30 |
| ssc04919 | Thyroid hormone signaling pathway | 48/1395 | 112/7674 | 9.57E-10 | 3.26E-09 | 1.40E-09 | 48 |
| ssc05219 | Bladder cancer | 24/1395 | 38/7674 | 9.95E-10 | 3.35E-09 | 1.44E-09 | 24 |
| ssc04137 | Mitophagy - animal | 33/1395 | 64/7674 | 1.33E-09 | 4.44E-09 | 1.91E-09 | 33 |
| ssc05225 | Hepatocellular carcinoma | 61/1395 | 160/7674 | 1.57E-09 | 5.15E-09 | 2.22E-09 | 61 |
| ssc04510 | Focal adhesion | 70/1395 | 194/7674 | 1.61E-09 | 5.22E-09 | 2.25E-09 | 70 |
| ssc04930 | Type II diabetes mellitus | 27/1395 | 47/7674 | 1.84E-09 | 5.92E-09 | 2.55E-09 | 27 |
| ssc04920 | Adipocytokine signaling pathway | 35/1395 | 71/7674 | 2.03E-09 | 6.46E-09 | 2.78E-09 | 35 |
| ssc04141 | Protein processing in endoplasmic reticulum | 61/1395 | 161/7674 | 2.08E-09 | 6.55E-09 | 2.82E-09 | 61 |
| ssc04662 | B cell receptor signaling pathway | 34/1395 | 70/7674 | 5.64E-09 | 1.74E-08 | 7.47E-09 | 34 |
| ssc04917 | Prolactin signaling pathway | 34/1395 | 70/7674 | 5.64E-09 | 1.74E-08 | 7.47E-09 | 34 |
| ssc05222 | Small cell lung cancer | 40/1395 | 91/7674 | 1.00E-08 | 3.05E-08 | 1.31E-08 | 40 |
| ssc05321 | Inflammatory bowel disease (IBD) | 31/1395 | 62/7674 | 1.11E-08 | 3.33E-08 | 1.43E-08 | 31 |
| ssc04915 | Estrogen signaling pathway | 51/1395 | 130/7674 | 1.13E-08 | 3.37E-08 | 1.45E-08 | 51 |
| ssc04371 | Apelin signaling pathway | 52/1395 | 134/7674 | 1.26E-08 | 3.68E-08 | 1.58E-08 | 52 |
| ssc04550 | Signaling pathways regulating pluripotency of stem cells | 52/1395 | 134/7674 | 1.26E-08 | 3.68E-08 | 1.58E-08 | 52 |
| ssc05144 | Malaria | 26/1395 | 48/7674 | 1.99E-08 | 5.75E-08 | 2.48E-08 | 26 |
| ssc05132 | Salmonella infection | 36/1395 | 80/7674 | 2.59E-08 | 7.41E-08 | 3.19E-08 | 36 |
| ssc04658 | Th1 and Th2 cell differentiation | 38/1395 | 88/7674 | 4.21E-08 | 1.19E-07 | 5.13E-08 | 38 |
| ssc05140 | Leishmaniasis | 31/1395 | 65/7674 | 4.58E-08 | 1.28E-07 | 5.53E-08 | 31 |
| ssc04062 | Chemokine signaling pathway | 62/1395 | 177/7674 | 5.02E-08 | 1.39E-07 | 6.00E-08 | 62 |
| ssc05202 | Transcriptional misregulation in cancer | 58/1395 | 166/7674 | 1.50E-07 | 4.13E-07 | 1.78E-07 | 58 |
| ssc04370 | VEGF signaling pathway | 27/1395 | 55/7674 | 1.60E-07 | 4.36E-07 | 1.88E-07 | 27 |
| ssc04664 | Fc epsilon RI signaling pathway | 29/1395 | 64/7674 | 4.91E-07 | 1.32E-06 | 5.70E-07 | 29 |
| ssc05014 | Amyotrophic lateral sclerosis (ALS) | 24/1395 | 48/7674 | 5.07E-07 | 1.36E-06 | 5.84E-07 | 24 |
| ssc05323 | Rheumatoid arthritis | 35/1395 | 85/7674 | 5.82E-07 | 1.54E-06 | 6.63E-07 | 35 |
| ssc04730 | Long-term depression | 27/1395 | 58/7674 | 6.24E-07 | 1.64E-06 | 7.05E-07 | 27 |
| ssc04120 | Ubiquitin mediated proteolysis | 48/1395 | 134/7674 | 7.73E-07 | 2.01E-06 | 8.66E-07 | 48 |
| ssc04611 | Platelet activation | 44/1395 | 120/7674 | 1.07E-06 | 2.75E-06 | 1.19E-06 | 44 |
| ssc05410 | Hypertrophic cardiomyopathy (HCM) | 32/1395 | 77/7674 | 1.38E-06 | 3.53E-06 | 1.52E-06 | 32 |
| ssc04215 | Apoptosis - multiple species | 18/1395 | 32/7674 | 1.49E-06 | 3.78E-06 | 1.63E-06 | 18 |
| ssc04022 | cGMP-PKG signaling pathway | 54/1395 | 162/7674 | 2.17E-06 | 5.43E-06 | 2.34E-06 | 54 |
| ssc04750 | Inflammatory mediator regulation of TRP channels | 37/1395 | 97/7674 | 2.58E-06 | 6.41E-06 | 2.76E-06 | 37 |
| ssc04623 | Cytosolic DNA-sensing pathway | 25/1395 | 55/7674 | 2.80E-06 | 6.91E-06 | 2.98E-06 | 25 |
| ssc04115 | p53 signaling pathway | 29/1395 | 69/7674 | 3.27E-06 | 8.00E-06 | 3.44E-06 | 29 |
| ssc03013 | RNA transport | 50/1395 | 149/7674 | 4.13E-06 | 1.00E-05 | 4.31E-06 | 50 |
| ssc05034 | Alcoholism | 49/1395 | 147/7674 | 6.36E-06 | 1.53E-05 | 6.58E-06 | 49 |
| ssc04670 | Leukocyte transendothelial migration | 39/1395 | 110/7674 | 1.10E-05 | 2.62E-05 | 1.13E-05 | 39 |
| ssc04630 | JAK-STAT signaling pathway | 49/1395 | 151/7674 | 1.46E-05 | 3.45E-05 | 1.48E-05 | 49 |
| ssc04622 | RIG-I-like receptor signaling pathway | 26/1395 | 63/7674 | 1.55E-05 | 3.64E-05 | 1.57E-05 | 26 |
| ssc04650 | Natural killer cell mediated cytotoxicity | 36/1395 | 100/7674 | 1.61E-05 | 3.75E-05 | 1.61E-05 | 36 |
| ssc04912 | GnRH signaling pathway | 33/1395 | 89/7674 | 1.78E-05 | 4.11E-05 | 1.77E-05 | 33 |
| ssc05030 | Cocaine addiction | 21/1395 | 47/7674 | 2.42E-05 | 5.53E-05 | 2.38E-05 | 21 |
| ssc04932 | Non-alcoholic fatty liver disease (NAFLD) | 48/1395 | 150/7674 | 2.68E-05 | 6.09E-05 | 2.62E-05 | 48 |
| ssc04150 | mTOR signaling pathway | 47/1395 | 150/7674 | 5.87E-05 | 0.000132297 | 5.70E-05 | 47 |
| ssc05143 | African trypanosomiasis | 16/1395 | 33/7674 | 6.70E-05 | 0.000149716 | 6.45E-05 | 16 |
| ssc05016 | Huntington disease | 56/1395 | 190/7674 | 8.18E-05 | 0.000181398 | 7.81E-05 | 56 |
| ssc04270 | Vascular smooth muscle contraction | 38/1395 | 116/7674 | 0.000103727 | 0.0002282 | 9.83E-05 | 38 |
| ssc01523 | Antifolate resistance | 15/1395 | 32/7674 | 0.000182898 | 0.000399305 | 0.000171949 | 15 |
| ssc04640 | Hematopoietic cell lineage | 30/1395 | 88/7674 | 0.000247389 | 0.000536009 | 0.000230817 | 30 |
| ssc04520 | Adherens junction | 25/1395 | 69/7674 | 0.000275865 | 0.000593214 | 0.000255451 | 25 |
| ssc04928 | Parathyroid hormone synthesis, secretion and action | 34/1395 | 105/7674 | 0.00030179 | 0.000641972 | 0.000276447 | 34 |
| ssc04666 | Fc gamma R-mediated phagocytosis | 28/1395 | 81/7674 | 0.000303029 | 0.000641972 | 0.000276447 | 28 |
| ssc05146 | Amoebiasis | 30/1395 | 92/7674 | 0.000586289 | 0.001232931 | 0.000530927 | 30 |
| ssc03450 | Non-homologous end-joining | 8/1395 | 13/7674 | 0.000631059 | 0.001317393 | 0.000567298 | 8 |
| ssc05416 | Viral myocarditis | 21/1395 | 58/7674 | 0.000840459 | 0.00174182 | 0.000750066 | 21 |
| ssc05206 | MicroRNAs in cancer | 64/1395 | 243/7674 | 0.000866266 | 0.001776901 | 0.000765173 | 64 |
| ssc05216 | Thyroid cancer | 15/1395 | 36/7674 | 0.000869812 | 0.001776901 | 0.000765173 | 15 |
| ssc05414 | Dilated cardiomyopathy (DCM) | 27/1395 | 82/7674 | 0.000915946 | 0.001857876 | 0.000800042 | 27 |
| ssc03440 | Homologous recombination | 16/1395 | 40/7674 | 0.001005091 | 0.002024338 | 0.000871725 | 16 |
| ssc05412 | Arrhythmogenic right ventricular cardiomyopathy (ARVC) | 23/1395 | 67/7674 | 0.001135693 | 0.002271387 | 0.000978109 | 23 |
| ssc04960 | Aldosterone-regulated sodium reabsorption | 15/1395 | 37/7674 | 0.001220329 | 0.00242371 | 0.001043703 | 15 |
| ssc04710 | Circadian rhythm | 13/1395 | 30/7674 | 0.001234325 | 0.002434599 | 0.001048392 | 13 |
| ssc05231 | Choline metabolism in cancer | 30/1395 | 96/7674 | 0.001280337 | 0.002508057 | 0.001080024 | 30 |
| ssc04925 | Aldosterone synthesis and secretion | 28/1395 | 91/7674 | 0.00236452 | 0.00460036 | 0.001981016 | 28 |
| ssc02010 | ABC transporters | 17/1395 | 47/7674 | 0.002591517 | 0.005007932 | 0.002156526 | 17 |
| ssc05020 | Prion diseases | 13/1395 | 33/7674 | 0.00345123 | 0.006624509 | 0.002852659 | 13 |
| ssc04310 | Wnt signaling pathway | 41/1395 | 153/7674 | 0.004983795 | 0.009502436 | 0.004091958 | 41 |
| ssc04744 | Phototransduction | 11/1395 | 27/7674 | 0.00517125 | 0.009794553 | 0.00421775 | 11 |
