# Supplementary material for: Prediction of antiviral drugs against African swine fever viruses based on protein–protein interaction analysis
Source: PeerJ. 2020 Apr 1;8:e8855. doi: 10.7717/peerj.8855 (PMC7127483; doi:10.7717/peerj.8855)
Supplement: Supplemental Information 5 [file peerj-08-8855-s005.doc]

Table S3. The predicted drugs targeting the ASFV-interacting swine proteins and ASFV proteins.

| **Part I: the predicted drugs targeting the ASFV-interacting swine proteins** | | | | | |
| --- | --- | --- | --- | --- | --- |
| **Swine protein (ID in STRING database)** | **Hits in Drugbank** | **ID for Drugs in Drugbank** | **Drug Type** | **Drug group** | **ATC code** |
| ENSSSCP00000001854 | P08238 | DB02424 | SmallMolecule | experimental investigational |  |
| ENSSSCP00000001854 | P08238 | DB02754 | SmallMolecule | experimental |  |
| ENSSSCP00000001854 | P08238 | DB03758 | SmallMolecule | experimental |  |
| ENSSSCP00000001854 | P08238 | DB05134 | SmallMolecule | investigational |  |
| ENSSSCP00000001854 | P08238 | DB06070 | SmallMolecule | investigational |  |
| ENSSSCP00000001854 | P08238 | DB07594 | SmallMolecule | experimental |  |
| ENSSSCP00000001854 | P08238 | DB07877 | SmallMolecule | experimental |  |
| ENSSSCP00000001854 | P08238 | DB08045 | SmallMolecule | experimental |  |
| ENSSSCP00000001854 | P08238 | DB08153 | SmallMolecule | experimental |  |
| ENSSSCP00000001854 | P08238 | DB08292 | SmallMolecule | experimental |  |
| ENSSSCP00000001854 | P08238 | DB08293 | SmallMolecule | experimental |  |
| ENSSSCP00000001854 | P08238 | DB08346 | SmallMolecule | experimental |  |
| ENSSSCP00000001854 | P08238 | DB08464 | SmallMolecule | experimental |  |
| ENSSSCP00000001854 | P08238 | DB08465 | SmallMolecule | experimental |  |
| ENSSSCP00000001854 | P08238 | DB09221 | SmallMolecule | experimental |  |
| ENSSSCP00000024932 | Q8IL88 | DB11638 | SmallMolecule | experimental investigational | P01BF05 |
| ENSSSCP00000013730 | P62136 | DB02506 | SmallMolecule | experimental |  |
| ENSSSCP00000000906 | P14625 | DB00615 | SmallMolecule | approved investigational | J04AB04 |
| ENSSSCP00000000906 | P14625 | DB02103 | SmallMolecule | experimental |  |
| ENSSSCP00000000906 | P14625 | DB02424 | SmallMolecule | experimental investigational |  |
| ENSSSCP00000000906 | P14625 | DB02935 | SmallMolecule | experimental |  |
| ENSSSCP00000000906 | P14625 | DB03719 | SmallMolecule | experimental |  |
| ENSSSCP00000000906 | P14625 | DB03758 | SmallMolecule | experimental |  |
| ENSSSCP00000000906 | P14625 | DB08464 | SmallMolecule | experimental |  |
| ENSSSCP00000000906 | P14625 | DB08465 | SmallMolecule | experimental |  |
| ENSSSCP00000000906 | P14625 | DB09130 | SmallMolecule | approved investigational |  |
| ENSSSCP00000024945 | Q13526 | DB01766 | SmallMolecule | experimental |  |
| ENSSSCP00000024945 | Q13526 | DB06867 | SmallMolecule | experimental |  |
| ENSSSCP00000016724 | P42574 | DB01017 | SmallMolecule | approved investigational | J01AA08 |
| ENSSSCP00000016724 | P42574 | DB03124 | SmallMolecule | experimental |  |
| ENSSSCP00000016724 | P42574 | DB05408 | SmallMolecule | investigational |  |
| ENSSSCP00000016724 | P42574 | DB06862 | SmallMolecule | experimental |  |
| ENSSSCP00000016724 | P42574 | DB07696 | SmallMolecule | experimental |  |
| ENSSSCP00000016724 | P42574 | DB08213 | SmallMolecule | experimental |  |
| ENSSSCP00000016724 | P42574 | DB08229 | SmallMolecule | experimental |  |
| ENSSSCP00000016724 | P42574 | DB08251 | SmallMolecule | experimental |  |
| ENSSSCP00000016724 | P42574 | DB08497 | SmallMolecule | experimental |  |
| ENSSSCP00000016724 | P42574 | DB08498 | SmallMolecule | experimental |  |
| ENSSSCP00000016724 | P42574 | DB08499 | SmallMolecule | experimental |  |
| ENSSSCP00000016724 | P42574 | DB13751 | SmallMolecule | approved experimental | A05BA08 |
| ENSSSCP00000014315 | P53041 | DB00171 | SmallMolecule | investigational nutraceutical |  |
| ENSSSCP00000014532 | Q13526 | DB01766 | SmallMolecule | experimental |  |
| ENSSSCP00000014532 | Q13526 | DB06867 | SmallMolecule | experimental |  |
| ENSSSCP00000008635 | P01584 | DB01017 | SmallMolecule | approved investigational | J01AA08 |
| ENSSSCP00000008635 | P01584 | DB05133 | SmallMolecule | investigational |  |
| ENSSSCP00000008635 | P01584 | DB05260 | SmallMolecule | approved investigational |  |
| ENSSSCP00000008635 | P01584 | DB05412 | SmallMolecule | investigational |  |
| ENSSSCP00000008635 | P01584 | DB05442 | SmallMolecule | investigational |  |
| ENSSSCP00000008635 | P01584 | DB05470 | SmallMolecule | investigational |  |
| ENSSSCP00000008635 | P01584 | DB05507 | SmallMolecule | investigational |  |
| ENSSSCP00000008635 | P01584 | DB05767 | SmallMolecule | investigational |  |
| ENSSSCP00000008635 | P01584 | DB06168 | Protein/Peptide | approved investigational | L04AC08 |
| ENSSSCP00000008635 | P01584 | DB06372 | Protein/Peptide | approved investigational | L04AC04 |
| ENSSSCP00000008635 | P01584 | DB10772 | Protein/Peptide | approved |  |
| ENSSSCP00000008635 | P01584 | DB11967 | SmallMolecule | approved investigational |  |
| ENSSSCP00000008635 | P01584 | DB12119 | Protein/Peptide | investigational |  |
| ENSSSCP00000008635 | P01584 | DB12140 | SmallMolecule | investigational |  |
| ENSSSCP00000009105 | P62136 | DB02506 | SmallMolecule | experimental |  |
| ENSSSCP00000010492 | P36873 | DB02169 | SmallMolecule | experimental |  |
| ENSSSCP00000010492 | P36873 | DB02860 | SmallMolecule | experimental |  |
| ENSSSCP00000010492 | P36873 | DB04738 | SmallMolecule | experimental |  |
| ENSSSCP00000001645 | Q07817 | DB07108 | SmallMolecule | experimental |  |
| ENSSSCP00000001645 | Q07817 | DB09401 | SmallMolecule | approved investigational |  |
| ENSSSCP00000001645 | Q07817 | DB13044 | SmallMolecule | investigational |  |
| ENSSSCP00000002743 | P07900 | DB00615 | SmallMolecule | approved investigational | J04AB04 |
| ENSSSCP00000002743 | P07900 | DB00716 | SmallMolecule | approved investigational | R01AC07 |
| ENSSSCP00000002743 | P07900 | DB02359 | SmallMolecule | experimental |  |
| ENSSSCP00000002743 | P07900 | DB02424 | SmallMolecule | experimental investigational |  |
| ENSSSCP00000002743 | P07900 | DB02550 | SmallMolecule | experimental |  |
| ENSSSCP00000002743 | P07900 | DB02754 | SmallMolecule | experimental |  |
| ENSSSCP00000002743 | P07900 | DB02840 | SmallMolecule | experimental |  |
| ENSSSCP00000002743 | P07900 | DB03093 | SmallMolecule | experimental |  |
| ENSSSCP00000002743 | P07900 | DB03137 | SmallMolecule | experimental |  |
| ENSSSCP00000002743 | P07900 | DB03504 | SmallMolecule | experimental |  |
| ENSSSCP00000002743 | P07900 | DB03749 | SmallMolecule | experimental |  |
| ENSSSCP00000002743 | P07900 | DB03809 | SmallMolecule | experimental |  |
| ENSSSCP00000002743 | P07900 | DB03899 | SmallMolecule | experimental |  |
| ENSSSCP00000002743 | P07900 | DB04054 | SmallMolecule | experimental |  |
| ENSSSCP00000002743 | P07900 | DB04216 | SmallMolecule | experimental investigational |  |
| ENSSSCP00000002743 | P07900 | DB04254 | SmallMolecule | experimental |  |
| ENSSSCP00000002743 | P07900 | DB04505 | SmallMolecule | experimental |  |
| ENSSSCP00000002743 | P07900 | DB04588 | SmallMolecule | experimental |  |
| ENSSSCP00000002743 | P07900 | DB05134 | SmallMolecule | investigational |  |
| ENSSSCP00000002743 | P07900 | DB06070 | SmallMolecule | investigational |  |
| ENSSSCP00000002743 | P07900 | DB06956 | SmallMolecule | experimental |  |
| ENSSSCP00000002743 | P07900 | DB06957 | SmallMolecule | experimental |  |
| ENSSSCP00000002743 | P07900 | DB06958 | SmallMolecule | experimental |  |
| ENSSSCP00000002743 | P07900 | DB06961 | SmallMolecule | experimental |  |
| ENSSSCP00000002743 | P07900 | DB06964 | SmallMolecule | experimental |  |
| ENSSSCP00000002743 | P07900 | DB06969 | SmallMolecule | experimental |  |
| ENSSSCP00000002743 | P07900 | DB07100 | SmallMolecule | experimental |  |
| ENSSSCP00000002743 | P07900 | DB07317 | SmallMolecule | experimental |  |
| ENSSSCP00000002743 | P07900 | DB07319 | SmallMolecule | experimental |  |
| ENSSSCP00000002743 | P07900 | DB07324 | SmallMolecule | experimental |  |
| ENSSSCP00000002743 | P07900 | DB07325 | SmallMolecule | experimental |  |
| ENSSSCP00000002743 | P07900 | DB07495 | SmallMolecule | experimental |  |
| ENSSSCP00000002743 | P07900 | DB07502 | SmallMolecule | experimental |  |
| ENSSSCP00000002743 | P07900 | DB07594 | SmallMolecule | experimental |  |
| ENSSSCP00000002743 | P07900 | DB07601 | SmallMolecule | experimental |  |
| ENSSSCP00000002743 | P07900 | DB07877 | SmallMolecule | experimental |  |
| ENSSSCP00000002743 | P07900 | DB08194 | SmallMolecule | experimental |  |
| ENSSSCP00000002743 | P07900 | DB08197 | SmallMolecule | experimental |  |
| ENSSSCP00000002743 | P07900 | DB08436 | SmallMolecule | experimental |  |
| ENSSSCP00000002743 | P07900 | DB08442 | SmallMolecule | experimental |  |
| ENSSSCP00000002743 | P07900 | DB08443 | SmallMolecule | experimental |  |
| ENSSSCP00000002743 | P07900 | DB08557 | SmallMolecule | experimental |  |
| ENSSSCP00000002743 | P07900 | DB08786 | SmallMolecule | experimental |  |
| ENSSSCP00000002743 | P07900 | DB08787 | SmallMolecule | experimental |  |
| ENSSSCP00000002743 | P07900 | DB08788 | SmallMolecule | experimental |  |
| ENSSSCP00000002743 | P07900 | DB08789 | SmallMolecule | experimental |  |
| ENSSSCP00000002743 | P07900 | DB09130 | SmallMolecule | approved investigational |  |
| ENSSSCP00000002743 | P07900 | DB09221 | SmallMolecule | experimental |  |
| ENSSSCP00000002743 | P07900 | DB12442 | SmallMolecule | investigational |  |
| ENSSSCP00000009781 | Q08209 | DB08231 | SmallMolecule | experimental |  |
| ENSSSCP00000021537 | Q13526 | DB01766 | SmallMolecule | experimental |  |
| ENSSSCP00000021537 | Q13526 | DB06867 | SmallMolecule | experimental |  |
| ENSSSCP00000003404 | Q07817 | DB07108 | SmallMolecule | experimental |  |
| ENSSSCP00000003404 | Q07817 | DB09401 | SmallMolecule | approved investigational |  |
| ENSSSCP00000003404 | Q07817 | DB13044 | SmallMolecule | investigational |  |
| ENSSSCP00000026450 | P61978 | DB11638 | SmallMolecule | experimental investigational | P01BF05 |
| ENSSSCP00000026450 | P61978 | DB12695 | SmallMolecule | investigational |  |
| ENSSSCP00000001491 | P01375 | DB00005 | Protein/Peptide | approved investigational | L04AB01 |
| ENSSSCP00000001491 | P01375 | DB00051 | Protein/Peptide | approved | L04AB04 |
| ENSSSCP00000001491 | P01375 | DB00065 | Protein/Peptide | approved | L04AB02 |
| ENSSSCP00000001491 | P01375 | DB00608 | SmallMolecule | approved investigational vet_approved | P01BA01 |
| ENSSSCP00000001491 | P01375 | DB00668 | SmallMolecule | approved vet_approved | S01EA01 |
| ENSSSCP00000001491 | P01375 | DB00852 | SmallMolecule | approved | R01BA52 |
| ENSSSCP00000001491 | P01375 | DB01041 | SmallMolecule | approved investigational withdrawn | L04AX02 |
| ENSSSCP00000001491 | P01375 | DB01296 | SmallMolecule | approved investigational | M01AX05 |
| ENSSSCP00000001491 | P01375 | DB01407 | SmallMolecule | approved investigational vet_approved | R03AC14 |
| ENSSSCP00000001491 | P01375 | DB01411 | SmallMolecule | investigational | R03DC02 |
| ENSSSCP00000001491 | P01375 | DB01427 | SmallMolecule | approved | C01CE01 |
| ENSSSCP00000001491 | P01375 | DB02325 | SmallMolecule | approved investigational | D08AX05 |
| ENSSSCP00000001491 | P01375 | DB04956 | Protein/Peptide | investigational | L04AB03 |
| ENSSSCP00000001491 | P01375 | DB05017 | SmallMolecule | investigational |  |
| ENSSSCP00000001491 | P01375 | DB05207 | SmallMolecule | investigational |  |
| ENSSSCP00000001491 | P01375 | DB05218 | Protein/Peptide | investigational |  |
| ENSSSCP00000001491 | P01375 | DB05303 | SmallMolecule | investigational |  |
| ENSSSCP00000001491 | P01375 | DB05412 | SmallMolecule | investigational |  |
| ENSSSCP00000001491 | P01375 | DB05470 | SmallMolecule | investigational |  |
| ENSSSCP00000001491 | P01375 | DB05513 | SmallMolecule | investigational |  |
| ENSSSCP00000001491 | P01375 | DB05676 | SmallMolecule | approved investigational | L04AA32 |
| ENSSSCP00000001491 | P01375 | DB05744 | SmallMolecule | investigational |  |
| ENSSSCP00000001491 | P01375 | DB05758 | Protein/Peptide | investigational |  |
| ENSSSCP00000001491 | P01375 | DB05767 | SmallMolecule | investigational |  |
| ENSSSCP00000001491 | P01375 | DB05869 | SmallMolecule | investigational |  |
| ENSSSCP00000001491 | P01375 | DB05879 | Protein/Peptide | investigational |  |
| ENSSSCP00000001491 | P01375 | DB05968 | SmallMolecule | investigational |  |
| ENSSSCP00000001491 | P01375 | DB05992 | SmallMolecule | investigational |  |
| ENSSSCP00000001491 | P01375 | DB06444 | SmallMolecule | investigational |  |
| ENSSSCP00000001491 | P01375 | DB06495 | Protein/Peptide | investigational |  |
| ENSSSCP00000001491 | P01375 | DB06674 | Protein/Peptide | approved | L04AB06 |
| ENSSSCP00000001491 | P01375 | DB08904 | Protein/Peptide | approved | L04AB05 |
| ENSSSCP00000001491 | P01375 | DB08910 | SmallMolecule | approved | L04AX06 |
| ENSSSCP00000001491 | P01375 | DB09221 | SmallMolecule | experimental |  |
| ENSSSCP00000001491 | P01375 | DB10770 | Protein/Peptide | approved |  |
| ENSSSCP00000001491 | P01375 | DB10772 | Protein/Peptide | approved |  |
| ENSSSCP00000001491 | P01375 | DB11967 | SmallMolecule | approved investigational |  |
| ENSSSCP00000001491 | P01375 | DB12140 | SmallMolecule | investigational |  |
| ENSSSCP00000001491 | P01375 | DB13751 | SmallMolecule | approved experimental | A05BA08 |
| ENSSSCP00000013805 | Q00653 | DB01296 | SmallMolecule | approved investigational | M01AX05 |
| ENSSSCP00000013805 | Q00653 | DB05212 | SmallMolecule | investigational |  |
| ENSSSCP00000013805 | Q00653 | DB05451 | SmallMolecule | investigational |  |
| ENSSSCP00000013805 | Q00653 | DB05464 | SmallMolecule | investigational |  |
| ENSSSCP00000013805 | Q00653 | DB05471 | Protein/Peptide | investigational |  |
| ENSSSCP00000013805 | Q00653 | DB05487 | SmallMolecule | investigational |  |
| ENSSSCP00000013805 | Q00653 | DB05767 | SmallMolecule | investigational |  |
|  |  |  |  |  |  |
| **Part II: the predicted drugs targeting the ASFV protein** | | | | | |
| **Accession number of ASFV protein in NCBI RefSeq database** | **Hits in Drugbank** | **ID for Drugs in Drugbank** | **Drug Type** | **Drug group** | **ATC code** |
| NP_042834 | P62837 | DB02418 | SmallMolecule | experimental |  |
| NP_042823 | Q8II92 | DB04685 | SmallMolecule | experimental |  |
| NP_042813 | Q86V86 | DB12010 | SmallMolecule | approved investigational |  |
| NP_042792 | P0A8T7 | DB00615 | SmallMolecule | approved investigational | J04AB04 |
| NP_042792 | P0A8T7 | DB01045 | SmallMolecule | approved | J04AM05 |
| NP_042792 | P0A8T7 | DB11753 | SmallMolecule | approved investigational | S02AA12 |
| NP_042749 | Q8N1G2 | DB00118 | SmallMolecule | approved investigational nutraceutical | A16AA02 |
| NP_042747 | Q8RQE9 | DB04788 | SmallMolecule | experimental |  |
| NP_042747 | Q8RQE9 | DB08226 | SmallMolecule | experimental |  |
| NP_042747 | Q8RQE9 | DB08266 | SmallMolecule | experimental |  |
| NP_042744 | P04183 | DB01692 | SmallMolecule | experimental |  |
| NP_042744 | P04183 | DB02452 | SmallMolecule | experimental |  |
| NP_042739 | P23921 | DB00242 | SmallMolecule | approved investigational | L01BB04 |
| NP_042739 | P23921 | DB00441 | SmallMolecule | approved | L01BC05 |
| NP_042739 | P23921 | DB00631 | SmallMolecule | approved investigational | L01BB06 |
| NP_042739 | P23921 | DB01005 | SmallMolecule | approved | L01XX05 |
| NP_042739 | P23921 | DB01073 | SmallMolecule | approved | L01BB05 |
| NP_042739 | P23921 | DB05003 | SmallMolecule | investigational |  |
| NP_042739 | P23921 | DB05420 | SmallMolecule | investigational |  |
| NP_042739 | P23921 | DB06433 | SmallMolecule | investigational |  |
| NP_042738 | P31350 | DB00242 | SmallMolecule | approved investigational | L01BB04 |
| NP_042738 | P31350 | DB05003 | SmallMolecule | investigational |  |
| NP_042738 | P31350 | DB05260 | SmallMolecule | approved investigational |  |
| NP_042738 | P31350 | DB05428 | SmallMolecule | investigational |  |
| NP_042738 | P31350 | DB05801 | SmallMolecule | investigational |  |
| NP_042729 | P23919 | DB01643 | SmallMolecule | experimental |  |
| NP_042729 | P23919 | DB03150 | SmallMolecule | experimental |  |
| NP_042729 | P23919 | DB03195 | SmallMolecule | experimental |  |
| NP_042729 | P23919 | DB03233 | SmallMolecule | experimental |  |
| NP_042729 | P23919 | DB03280 | SmallMolecule | experimental |  |
| NP_042729 | P23919 | DB03666 | SmallMolecule | experimental |  |
| NP_042729 | P23919 | DB03845 | SmallMolecule | experimental |  |
| NP_042729 | P23919 | DB04395 | SmallMolecule | experimental |  |
